# Cerebellar Electrical Activity as a Marker for Predicting Brain Health and Disease: A Review

**DOI:** 10.3390/brainsci16070758

**Published:** 2026-07-19

**Authors:** Gordana Stojadinović, Ljiljana Martać, Srđan Kesić, Branka Petković, Jelena Podgorac Kojadinović

**Affiliations:** Department of Neurophysiology, Institute for Biological Research “Siniša Stanković”—National Institute of the Republic of Serbia, University of Belgrade, Despota Stefana Blvd. 142, 11108 Belgrade, Serbia

**Keywords:** cerebellum, adaptive responses, spatio-temporal patterns, electrocerebellography, animal models

## Abstract

**Highlights:**

**What are the main findings?**

- Recent scientific research suggests that the cerebellum may play a significant role in understanding brain function in health, including sleep, motor activity, and social cognition, as well as in altered states and diseases such as anesthesia, epilepsy, traumatic brain injury, neurodegenerative diseases, and neuropsychiatric disorders.
- Rhythmic EEG changes in cerebellar electrical activity reflect functional connectivity between the cerebellum and the rest of the brain in both health and disease.

**What are the implications of the main findings?**

- Ongoing research on the cerebellum’s rhythmic patterns opens a window onto a broader understanding of neuronal network plasticity, shedding light on both healthy and pathological physiological conditions.
- The use of advanced EEG and fMRI metrics, along with more traditional spectral analysis, paves the way for a sophisticated understanding of the subtleties of cerebellar dynamics and its association with cerebellar functional networks across a myriad of functional states.

**Abstract:**

The cerebellum is traditionally considered a structure responsible for motor control, but it is also involved in auditory perception, vocalization, speech, memory, emotional control, and social cognition. Due to its high intrinsic synaptic plasticity and complex connectivity with other brain regions, it is of potential interest for monitoring adaptive responses under various physiological and pathological conditions to capture global brain dynamics. Nevertheless, the use of electrocerebellography (ECeG) to detect changes in cerebellar electrical activity is limited, and a systematic evaluation of ECeG data to inform future research directions is lacking. This review summarizes recent ECeG research to explore the contribution of this time-honored method to deciphering the cerebellum’s spatial and temporal dynamics in health and disease. ECeG studies from the past three decades examining the slow and fast adaptive responses of the cerebellum in different cerebellar layers during sleep, anesthesia, brain injury, epilepsy, neurodegenerative diseases, and neuropsychiatric disorders are compiled from the PubMed, Scopus, and Google Scholar databases and discussed accordingly. It can be concluded that, despite certain limitations, ECeG is a practical, valuable, and reliable technique for detecting and predicting the complex spatial and temporal features of cerebellar electrical activity.

## 1. Introduction

The cerebellum is traditionally regarded as a structure responsible for motor control, but it is also involved in non-motor functions such as auditory perception, attention, vocalization, speech, memory, emotional regulation, and social cognition [1,2,3,4]. Its primary role is to control motor function, a function first documented in [5]. Alongside its well-known motor functions, such as posture, vestibular activity, planning, coordination, timing, and the synergy of complex movements, its non-motor functions have recently attracted attention [4]. Owing to its high intrinsic synaptic plasticity and complex connectivity with other brain regions, monitoring its adaptive responses under various physiological and pathological conditions is of interest for understanding global brain dynamics [1]. For instance, a recent research direction that is gaining increasing attention concerns the role of the cerebellum in the pathology of psychiatric disease. Indeed, it may be implicated in insufficient emotional control, autism-like disorders, schizophrenia, bipolar and depressive disorders, and social disabilities [6,7]. Across the brain and medical conditions described in the following sections of this paper—motor control, social cognition, sleep, anesthesia, epilepsy, traumatic brain injury, neurodegeneration, and affective and emotional control—evidence points to a crucial role for the cerebellum, highlighting the need for further research. Although these associations are promising, our current understanding of cerebellar context-related function remains incomplete.

**Figure 1:**
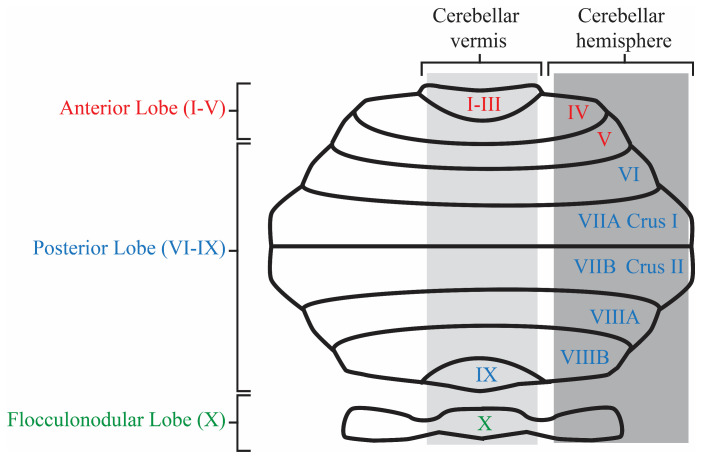
Schematic representation of the major anatomical subdivisions of the cerebellum. Created in Adobe Illustrator Version 2019 (23.1.0) by the authors.

**Figure 2:**
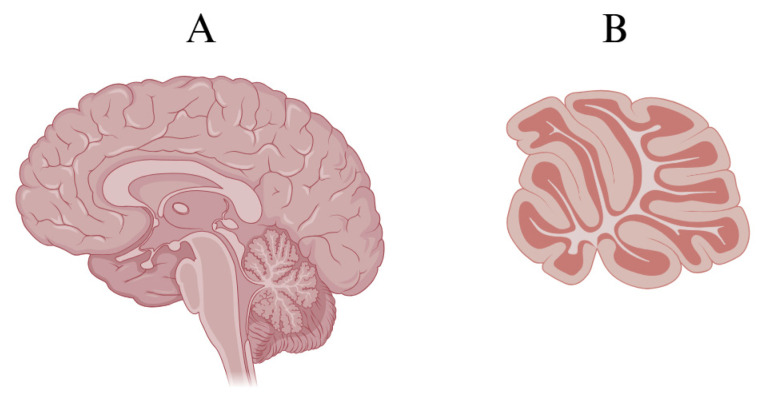
(**A**): Midsagittal section of the human brain. (**B**): Anatomy of cerebellar layers. The content of this figure is taken from BioRender. For the Department of Neurophysiology, Institute for Biological Research, the license is issued to Nataša Todorović for 2026.

Anatomically, the cerebellum can be divided into three major lobes: the anterior lobe (above the primary fissure); the posterior lobe (below the primary fissure); and the flocculonodular lobe (which is known as the phylogenetically oldest part), vestibulocerebellum or archicerebellum (below the posterior fissure) (Figure 1) [8]. Mediolaterally, the central part of the cerebellum is called the vermis (spinocerebellum or paleocerebellum), while the lateral regions are known as the hemispheres (cerebrocerebellum or neocerebellum). The hemispheres are the largest cerebellar region in mammals [9]. A cross-section of the cerebellum shows an outer, compactly folded layered cortex and an inner white matter region containing the deep cerebellar nuclei (fastigial, interpositus, and dentate nuclei). The cerebellar cortex has a three-layer structure (Figure 2): the molecular layer, the Purkinje cell layer, and the granule cell layer [10]. The outermost layer of the cerebellar cortex is the molecular layer, which contains the flattened dendritic trees of Purkinje cells and the extensive parallel fibers from the granule cell layer. The molecular layer also contains two types of inhibitory interneurons: stellate cells and basket cells. Both stellate and basket cells form GABAergic synapses on Purkinje cell dendrites [11].

Climbing and mossy fibers are two major primary afferents of the cerebellum [12]. The afferent cerebellar pathways involve sensory afferent systems (proprioceptors, visual, auditory, and vestibular information), cortico-cerebral afferents (cerebro-ponto-cerebellar pathway), and other cerebellar afferents (including the inferior olivary nucleus, red nucleus, vestibular system, reticular formation, nucleus locus ceruleus, and hypothalamic afferents). The axons of Purkinje cells and cerebellar nuclear neurons form efferents from the cerebellar cortex and cerebellum to other brain structures. The fastigial nucleus sends projections to the vestibular nucleus, the reticular formation, the inferior olivary nucleus, the superior colliculus, and to the thalamic nucleus; the nucleus interpositus sends divergent outputs to the red nucleus (magnocellular part), the periaqueductal gray matter, and the thalamus; and the dentate nucleus is connected via efferent fibers to the red nucleus (parvocellular part), the “motor” thalamic nucleus, and the intralaminar thalamic nucleus [11].

Purkinje cells have large spherical bodies and are arranged in a single-cell-thick layer of the cerebellar cortex, known as the Purkinje layer. Each Purkinje cell receives more synaptic inputs than any other cell type in the brain. After giving off collaterals, their axons project to the deep cerebellar nuclei. Purkinje cells use GABA as their neurotransmitter and therefore exert inhibitory effects on their targets. Granule cells of the cerebellum are among the smallest neurons in the brain. Their cell bodies are densely packed in a thick layer at the base of the cerebellar cortex. Granule cells receive excitatory input from mossy fibers and inhibitory input from Golgi cells. The thin, unmyelinated axons of granule cells ascend vertically to the upper (molecular) layer of the cortex, where they bifurcate, with each branch running horizontally to form a parallel fiber. Granule cells use glutamate as their neurotransmitter and therefore exert excitatory effects on their targets. Purkinje cells also receive input from the inferior olivary nucleus on the contralateral side of the brainstem via climbing fibers. A climbing fiber gives off collaterals to the deep cerebellar nuclei before entering the cerebellar cortex [11].

The current methodological inventory available for fundamental research and clinical diagnosis of cerebellar function/dysfunction has its limitations. Most data on the cerebellum come from histological sections, magnetic resonance imaging (MRI), and positron emission tomography (PET) [8]. However, electrocerebellography (ECeG) is less commonly used to assess cerebellar function. ECeG can be obtained by placing a recording electrode directly on the cerebellum, for example, during neurosurgical procedures or neurostimulation in human medicine [13]. ECeG studies of the human cerebellum are underrepresented; in vivo experimentation in small mammals is a more suitable approach for recording electrical signals from the cerebellum [14]. Adrian (1935) and Dow (1938) were the first to describe electrical waves in the cerebellum and their function [15,16]. Eccles (1967) described the cerebellum as a neuronal machine with circuits that control movements [17]. Soon after, Marr (1969) and Albus (1971) postulated that climbing fibers provide a teaching signal that modifies the parallel fiber–Purkinje cell synapses, allowing motor activity to be fine-tuned [18,19]. Studies by Llinás and Sugimori (1980), who focused on the electrophysiology of Purkinje neurons, contributed significantly to understanding the synaptic organization of the cerebellum and the basis of cerebellar diseases [20]. Finally, Ito (2000) expanded our understanding of cerebellar physiology, postulating that long-term depression (LTD) is the primary molecular and cellular mechanism of learning in the cerebellum [21].

Over the past three decades, our laboratory has provided insights into the adaptive oscillatory responses of the cerebellum under various physiological and pathological in vivo conditions, primarily using animal models of traumatic brain injury and different types of anesthesia [22,23,24,25,26,27,28]. Based on our longstanding and humble work in this field, we aim to summarize the limited literature on the use of ECeG to measure cerebellar electrical activity and to discuss other relevant findings on cerebellar electrical activity in relation to other neuroimaging techniques. We used PubMed, Scopus, and Google Scholar to collect relevant research findings, which were then critically evaluated and discussed. We selected a small but relevant body of literature data to discuss several topics addressing cerebellar electrical activity, which are presented in the following sections: (2) Cerebellum: From Motor Control to Social Cognition, (3) Role of the Cerebellum in Sleep, (4) Effects of General Anesthesia on Cerebellar Dysfunction, (5) Cerebellum and Epilepsy, (6) Effects of Traumatic Brain Injury on the Cerebellum, (7) Cerebellum and Neurodegenerative Diseases, and (8) Cerebellum and Neuropsychiatric Disorders. Some of the findings on the cerebellum’s vital role as a dynamic neurophysiological orchestrator are summarized in Table 1.

## 2. Cerebellum: From Motor Control to Social Cognition

The cerebellum receives afferent projections from the locus coeruleus (LC) and inferior olive (IO) in the form of mossy and climbing fibers. The LC and IO are part of a somatosensory system that modulates the electrical activity of Purkinje neurons, increasing their activity in response to significant stimuli and decreasing it when the stimuli are absent or irrelevant. Via norepinephrine and glutamate released at the ends of these axons, LC and IO are involved with the cerebellum in the control of posture and balance, motor coordination, and motor learning. Stimulation of LC increases the inhibitory effect of GABA on Purkinje cells, thereby inhibiting their activity [38]. The climbing fibers of IO provide strong excitatory input to the cerebellum, generating complex-spike-evoked postsynaptic potentials in Purkinje cells and playing a central role in motor control [39,40]. The increase in cerebellar activity also indicates synchronization between the cerebellum and other brain structures [41].

The oscillatory activity of specific layers in the cerebellar cortex, including their frequencies and functions, has been studied for decades. In the rat cerebellar granular layer of Crus II, theta oscillatory activity at 7–8 Hz is typical during immobility [29]. These oscillations cease with any movement of the animal. They are thought to reflect somatosensory system activity, which is critical for the cerebellum’s temporal encoding of incoming sensory data. Granule cells in acute cerebellar slices from rat pups exhibited theta-frequency bursting at 3–12 Hz [30]. These oscillations may be essential for synchronization, rhythmicity, and learning in the cerebellum. Oscillations at 13–18 Hz in local field potentials (LFPs) were observed in the cerebellar cortex of monkeys when the animals were immobile but awake and ready to respond to an auditory stimulus [31]. These oscillations originated in the granular cell layer and were particularly pronounced in the paramedian lobule of the cerebellum. These findings suggest that the cerebellum may be involved in higher-level neuronal integration, particularly in complex sensorimotor behavior. Cerebellar LFP oscillations of 13–25 Hz were recorded in the monkey paramedian lobule while the animals quietly awaited a reward after the appearance of a stimulus or during movement in response to a stimulus [32]. This study provides electrophysiological evidence for a specific pattern of cerebellar activity during anticipated events.

Action potentials recorded from the surface of the cerebellum have smaller amplitude and higher frequency than those from the cerebrum [16]. As recording electrodes penetrate deeper into the cerebellar layers, action potentials become even smaller in amplitude and higher in frequency. Stojadinović et al. [27] demonstrated that increasing the depth of ECeG recording electrodes through the cerebellar layers alters the spectral profiles of rat cerebellar electrical signals. The study showed increased participation in both the slowest delta (0.1–4.0 Hz) and the fastest gamma (32.1–128 Hz) frequency ranges. In this context, the impact of anesthesia on results must also be considered, as a slow-delta frequency range is observed in cortical layers under Nembutal anesthesia [27]. Other studies have reported the prevalence of lower gamma rhythm (30–80 Hz) and very fast oscillations (80–160 Hz) in the cerebellar cortex [33]. The role of these high-frequency rhythms may be in spatio-temporal coding between the cortex and cerebellum, that is, in the correct selection and execution of motor commands. Moreover, it was found that Purkinje cell activity is synchronized by a high-frequency (~200 Hz) oscillation. This activity is crucial for precise timing, which is essential for controlling complex, coordinated, and rapid movements [34]. Further research on the cerebellum is required for establishing a comprehensive understanding of the complex neuronal circuitry in the cerebellar cortex and the function of cerebellar rhythms.

## 3. Role of the Cerebellum in Sleep

Sleep is an important brain function. It supports cognitive processes, such as memory, learning, attention, language processing, decision-making, and even creativity [42,43,44,45,46,47]. Sleep is a state of reduced mental and physical activity in which consciousness is altered, and certain sensory activity is inhibited [48]. Sleep occurs in recurring periods, during which the body alternates between two distinct states: rapid-eye-movement (REM) and non-REM sleep [49]. Non-REM sleep occurs first and is called slow-wave sleep or deep sleep. The American Academy of Sleep Medicine (AASM) divides NREM into three stages: N1, N2, and N3 [50]. There are distinct electroencephalographic and other characteristics seen in each stage. In the N1 stage, alpha waves disappear, and theta waves appear. In the N2 stage, EEG recordings tend to show characteristic “sleep spindles”, which are short bursts of high-frequency brain activity, and “K-complexes” during this stage [51]. During the N3 stage, delta waves appear. The whole period normally proceeds in the order: N1, N2, N3, N2, and REM. Electroencephalography during REM sleep shows fast, low-amplitude, desynchronized neural oscillations that resemble the pattern seen during wakefulness, which differ from the slow-delta-wave pattern of NREM deep sleep [52]. An important element of this contrast is the 3–10 Hz (theta) rhythm in the hippocampus and 40–60 Hz (gamma) waves in the cortex [53,54].

The cerebellum is a brain region involved in both motor and non-motor processing, as well as in regulating sleep–wake cycles [55]. The presence of clock genes in the cerebellar cortex, as well as circadian oscillations in Purkinje cell firing rate, has been established [35]. The medial parabrachial nucleus (MPB) participates in sleep transitions between REM and NREM sleep [36,56]. Purkinje cells of lobules VIII–X project their axons directly to the ipsilateral MPB. These findings suggest that lobules VIII–X may affect MPB’s neural activity to regulate the sleep–wake cycle. Two coupled systems are involved in sleep neurophysiology, and information flows via two pathways: a directional flow from the motor cortex to the cerebellum during slow waves (consistent with their neocortical origin) and a reversal from the cerebellum to the thalamus and neocortex during spindle events. Since sleep spindles are implicated in the offline consolidation of procedural learning, it can be concluded that the cerebellum is substantially involved, via cerebelo-thalamo-neocortical communication, in sleep regulation [57]. Cerebellar activity during sleep reflects the state of the neocortex [58]. In general, there is decreased cerebellar activity during NREM sleep compared with that during wakefulness [59]. During the N1 stage of NREM, cerebellar signals are lower compared to those during wakefulness. During the N2 stage, changes in cerebellar electrical activity co-occur with K-complexes and neocortical sleep spindles [60,61]. Finally, during N3, cerebellar electrical activity co-occurs with neocortical slow waves [62].

On the contrary, both the cerebellar hemispheres and vermis show increased activity during REM sleep [63]. The changes in the activity of the cerebellum during the sleep–wake cycle reflect changes in its afferent glutamatergic excitatory inputs from the mossy- and climbing-fiber systems [64]. Mossy and climbing fibers modulate Purkinje cell excitatory responses and contribute to the cell’s overall activity [65]. Climbing fibers originate from the inferior olive and form a powerful, one-to-one synapse with a single Purkinje cell. Each climbing fiber makes numerous contacts along the Purkinje cell’s dendrites, releasing glutamate that activates both ionotropic (AMPA) and metabotropic (mGluR1) glutamate receptors. This input evokes a strong, complex spike response in the Purkinje cell [39]. Parallel fibers, on the other hand, originate from granule cells in the cerebellar cortex and form a much larger number of synapses with each Purkinje cell, potentially in the range of 100,000 to 1,000,000. These fibers release glutamate, primarily activating AMPA receptors, which generate smaller, “simple spike” responses in the Purkinje cell. The parallel fiber input is crucial for modulating Purkinje cell activity and for contributing to motor coordination and learning [66]. Complex spike activity generated by the climbing-fiber input is lowest in REM sleep stage and may be compatible with the loss of muscle tone during this stage [37,67]. Simple spike activity generated by the parallel fiber input to the Purkinje cell is increased during REM sleep [68]. In addition, studies have shown reduced firing activity in the deep cerebellar nuclei (fastigial and interpositus) during the transition from quiet wakefulness to slow-wave sleep, while increased firing activity occurs during REM sleep [69].

The hippocampus also plays a crucial role in sleep regulation by interacting with the cerebellum and modulating cerebellar activity. It was found that delta oscillations coordinate intracerebellar and cerebello-hippocampal network dynamics during sleep. During non-REM sleep, prominent delta-frequency coherence was observed between the cerebellum and the hippocampus. Moreover, sharp-wave ripple activity of the hippocampus evoked discrete LFP modulation in all recorded cerebellar regions. During REM sleep, cerebellar delta phasic sharp potentials (PSPs) modulated hippocampal potentials. Likewise, within the cerebellum, prominent LFP oscillations were observed at both low delta (4 Hz) and gamma (250 Hz) frequencies during non-REM and REM sleep [70].

Cerebellar lesions can significantly affect sleep patterns and may lead to sleep disorders. Damage to the cerebellum can alter sleep architecture, including increased daytime sleepiness, disrupted REM sleep, and difficulty initiating or maintaining sleep [71]. Individuals with cerebellar ataxia, a condition characterized by impaired coordination and balance, often experience sleep disturbances, including excessive daytime sleepiness and REM sleep abnormalities [62]. Neurodegenerative disorders affecting the cerebellum, such as Alzheimer’s and Parkinson’s disease, are frequently associated with sleep problems like REM sleep behavior disorder, insomnia, and sleep apnea [72,73].

Although the precise role of the cerebellum in regulating sleep remains to be further investigated, it is clear that its primary role is in motor learning and cognitive processes [74]. Malfunctions of the cerebellum lead to changes in sleep, but not to abolishment of sleep [75]. The contribution of the cerebellum to sleep disturbances should be further elucidated. Further investigations involving multiple single-unit recordings of both cerebellar and extracerebellar neurons during various sleep stages are necessary to evaluate cerebellar physiology and its interactions with other cortical and subcortical structures. Some principal findings on the cerebellar role in neurologic disorders are summarized in Table 2.

## 4. Effects of General Anesthesia on Cerebellar Dysfunction

Postoperative sleep disturbances frequently occur after general anesthesia. It was reported that sleep patterns were disturbed on the first night after surgery, with reduced total sleep time, slow-wave sleep (SWS), and REM sleep [93]. Sleep interruptions may last for three to four nights or longer, up to several weeks after surgery [94]. General anesthesia can affect the cerebellum, altering its activity and potentially affecting motor and non-motor functions [95]. Specifically, anesthetics can disrupt the delicate balance of neuronal communication in the cerebellum, potentially affecting processes such as sleep–wake cycles and cognitive functions [76,77]. During recovery from general anesthesia, signs of cerebellar dysfunction may appear, including mild to severe ataxia of all four extremities, intentional tremor, absent menace response, and delayed hopping [96]. Anesthetics induce widespread cell death, permanent neuronal deletion, and neurocognitive impairment in immature animals, raising substantial concerns about similar effects occurring in young children. Decreases in IQ, language comprehension, and the volume of the cerebellar cortex have been noted following childhood surgery under anesthesia [97]. Postoperative sleep deprivation may disrupt functional connectivity between the lateral cerebellar nuclei and the neocortical superior temporal sulcus [43]. The cerebellum also participates in conscious recovery from anesthesia. Cerebellar Purkinje cell firing facilitates conscious recovery from the anesthetic state by coordinating neuronal communication with the motor cortex [78].

Propofol is commonly used for intravenous induction of general anesthesia in the clinic. Propofol behaves as a GABAA receptor agonist [98]. It may cause neurotoxicity in the developing brain [99]. Exposure to propofol resulted in a significant reduction in Purkinje cells and their dendrite length [100]. Propofol depresses cerebellar Purkinje cell activity by activating GABAA and glycine receptors [101]. Via the climbing fiber–Purkinje cell pathway and NMDA receptors, propofol affects information processing, inducing cerebellar dysfunction and movement disorders [102]. Ketamine is a general anesthetic and NMDA receptor antagonist with analgesic and hallucinogenic properties, used medically for anesthesia, depression, and pain management [103]. In cerebellar oscillatory activity, ketamine anesthesia causes the prominent presence of slow-delta (0.1–4.0 Hz) and theta (4.1–8.0 Hz) frequency ranges, the same as on the cerebrum [25]. Nembutal (Pentobarbital) is a short-acting barbiturate typically used as a sedative, as a preanesthetic, and to control convulsions in emergencies. Like other barbiturates, nembutal binds to the GABA-A receptor and increases the duration of ion-channel opening [104]. In the cerebellum, nembutal anesthesia causes the prominent presence of slow-delta (0.1–4.0 Hz) and theta (4.1–8.0 Hz) frequency oscillations [25,105].

Diethyl ether was one of the earliest and most widely used general anesthetics [106]. Although effective, it has been largely replaced by safer, better-controlled agents due to its flammability and side effects [107]. The effect of ether anesthesia is based on a reversible loss of consciousness resulting from its interaction with the central nervous system. The exact mechanism of ether anesthesia is not fully understood, but it has been shown to act on GABA-A receptors, glycine receptors, and other ion channels, thereby increasing neurotransmission inhibition and reducing neuronal activity. The electrocortical dynamics of the cerebellum have shown the prominent presence of slow-delta (0.1–4.0 Hz) and theta (4.1–8.0 Hz) frequency oscillations during the first 30 min after consciousness is restored from diethyl-ether anesthesia [28].

Sevoflurane is an inhalational anesthetic, used for induction and maintenance of general anesthesia [108]. Sevoflurane acts as a positive allosteric modulator of the GABAA receptor [109]. However, it also acts as an NMDA receptor antagonist, potentiates glycine receptor currents, and inhibits nAChR and 5-HT3 receptor currents [110,111,112]. Sevoflurane anesthesia disrupts functional connectivity in cerebellar circuits due to neuronal loss, particularly in the molecular and Purkinje cell layers [113]. Repeated inhalation of sevoflurane impairs Purkinje cell information transmission and delays motor development via the GABAA receptor [114].

Xylazine anesthesia has veterinary applications for sedation, anesthesia, muscle relaxation, and analgesia in animals [115]. Xylazine acts as an α2 adrenergic receptor agonist [116]. There is a differential effect of ketamine/xylazine anesthesia on cerebral and cerebellar cortical activity. Ketamine/xylazine anesthesia, which contrasts with motor cortex anesthesia, suppresses most activity in the cerebellar cortex [117]. Zoletil is a combination of tiletamine hydrochloride (an NMDA receptor antagonist) and zolazepam hydrochloride (a benzodiazepine, which is a GABA receptor agonist) used as a veterinary anesthetic [118,119,120]. In the cerebellar electrical activity, the dominant presence of slow-delta (0.1–4.0 Hz) and theta (4.1–8.0 Hz) frequency bands was observed during zoletil anesthesia [25]. Urethane is also a general anesthetic widely used in animal research [121]. It potentiated the functions of nACh, GABA, and glycine receptors, and inhibited NMDA and AMPA receptors [122]. Urethane anesthesia induced more prominent neuronal oscillations in both Purkinje cells and Golgi cells than in the wake state [123].

Anesthesia undoubtedly affects the neuronal circuitry in the cerebellar cortex. But it is not fully understood how anesthesia affects the network dynamics of the cerebellum and its overall function. Additional research data could provide a new framework for exploring the influence of anesthesia on cerebellar activity, cerebellar dysfunction, and movement disorders.

## 5. Cerebellum and Epilepsy

There is evidence that the cerebellum is involved in seizures, and cerebellar impairments have been observed in patients with epilepsy [79]. Cerebellar degeneration was most prevalent in patients with temporal lobe epilepsy and in patients using phenytoin therapy [80]. Cerebellar atrophy and loss of cerebellar volume were noted among patients with chronic epilepsy [124]. Seizures arise from the cerebral cortex, glossing over infratentorial structures such as the cerebellum, which are believed to modulate rather than generate seizures [81]. The ventral posteromedial nucleus (VPM) of the thalamus is a source of generalized convulsive epileptic activity, and cerebellar and cerebral cortical afferents to the thalamus are important contributors to the seizure [125]. The cerebellum projects to numerous thalamic nuclei. Electrophysiological recordings revealed that cerebellar stimulation resulted in enhanced action potential firing in thalamic nucleus neurons [126]. Significant reductions in cerebellar, total brain, and gray matter volumes were found after MRI in patients with epilepsy [127]. Changes in the cerebellum are routinely observed in epilepsy, including impaired structural connectivity, volume changes, alterations in cerebellar blood flow, Purkinje cell atrophy, and increased metabolic activity [128,129]. The medial temporal lobe, occipitotemporal areas, cerebellum, cingulate cortex, insula, and thalamus are more likely to be atrophied in patients with epilepsy [130]. A cerebellar abscess can alter the electrical activity of the cerebral cortex, manifesting as ictal phenomena on EEG recordings [131]. Injury of the cerebellum enhanced the electrocortical manifestations of experimental epilepsy, while cerebellar stimulation leads to inhibition of epileptic waves [132,133]. Cerebellar optogenetic intervention inhibited spontaneous hippocampal seizures in a mouse model of temporal lobe epilepsy [134]. In addition, decreased cerebellar functional connectivity with the putamen and motor cortex was also observed in epilepsy [135].

Patients with treatment-resistant epilepsy have more severe cerebral and cerebellar damage. There is a presence of cerebellar atrophy in epileptic patients with long-term phenytoin exposure, and other voltage-gated sodium channel blockers can cause overdose, neurotoxicity, and ataxia [136,137]. The age of patients also represents a significant factor that increases the occurrence of cerebellar damage in epileptic patients [138]. Published data showed that the cerebellum contributes to the genesis of epilepsy together with other brain structures. Synchronous spike activity in the cerebellum was noted during cerebral paroxysmal activity, but was less prominent than the spike discharges on the scalp [139]. Volumetric MRI and resting-state fMRI showed increased low-frequency oscillations in the thalamus, cortex, and cerebellar vermis in patients with epilepsy [140]. Magnetoencephalography showed paroxysmal activity in the cerebellar vermis and hemispheres in patients with epilepsy [82].

Furthermore, contralateral activation of the cerebellum was shown 14 s after ictal onset on the motor cortex [83]. Electrical neurostimulation is commonly applied in patients with drug-resistant epilepsy and may be outside the epileptic focus. Emerging data from various epilepsies indicate that controlling the cerebellar output can be an effective way to stop seizures. By selectively increasing or decreasing the impulse activity of cerebellar neurons and the firing of thalamocortical neurons, it can effectively modulate epileptic activity [141]. Cerebellar stimulation induces prolonged cortical focal discharges in the forebrain, driven by GABA-ergic extrasynaptic inhibition within a thalamocortical network [142]. Phase-locked bursting of cerebellar units during high-voltage spindle is independent of rhythmic movement. Rhythmic output from the cerebellum may contribute to the maintenance of generalized petit mal seizures [143].

## 6. Effects of Traumatic Brain Injury on the Cerebellum

A traumatic brain injury (TBI), also known as an intracranial injury, is an injury to the brain caused by an external force [144]. TBI is graded as mild, moderate, or severe based on the level of consciousness, as measured by the Glasgow Coma Scale (GCS). Mild TBI is in most cases a concussion, and there is full neurological recovery, although many of these patients have short-term memory and concentration difficulties. In moderate TBI, the patient is lethargic or stuporous, and in severe injury the patient is comatose, unable to open his or her eyes or follow commands [145]. Damage from TBI can be focal or diffuse, limited to specific areas or distributed more broadly [146]. Some of the components of TBI, including ataxia, postural instability, tremor, impairments in balance and fine motor skills, and even cognitive deficits, can be attributed to cerebellar damage [147,148,149,150,151,152]. There is increasing interest in the effects of brain injury on the cerebellum. Even when not directly damaged, the cerebellum is of particular interest for understanding adaptive responses to brain injury, given its high intrinsic synaptic plasticity and complex connectivity with neocortical regions [153].

Traumatic brain injury is followed by degeneration of neurons and axons in the cerebellum, a decrease in gray matter thickness, activation of microglia and macrophages, and increased neurotransmission from mossy fibers to granule neurons and from granule neurons to Purkinje neurons [154,155]. Neurological damage does not occur immediately at the moment of impact (primary injury) but through delayed effects (secondary injury). Secondary brain injury is the leading cause of in-hospital deaths after traumatic brain injury [156]. Most secondary brain injury is caused by brain swelling, with an increase in intracranial pressure and a subsequent decrease in cerebral perfusion leading to ischemia [157]. Fluid percussion injury (FPI) is an established experimental model for studying secondary effects of TBI in animals [158]. Fluid percussion injury of the cerebellum leads to presynaptic hyperexcitability at cerebellar synapses and an increase in the amplitude of spikes from presynaptic mossy fibers [159]. Electrophysiological monitoring of fluid percussion injury of the cerebellum showed a decreasing amplitude of evoked potentials in the paramedian lobule of the posterior cerebellum as a result of disruption in the flow of sensory information. Decreases in amplitude were notably the largest, ~50% and 24%, during the first 24 h in the acute period and the 3–7-day period, respectively.

Furthermore, immunohistochemical analysis of the cerebellum showed Purkinje cell loss [160]. Disrupted white matter integrity of the cerebellar peduncle and decreased gamma frequency band (25–40 Hz) synchronization in EEG were observed in patients with mild traumatic brain injury [161]. Generally, reduced spontaneous cerebellar electric activity was present in acute traumatic brain injury patients [84]. The consequences of traumatic brain injury can appear on the cerebellum even when it is not directly affected by the injury. Injury of the cerebral cortex is followed by a decrease in the presence of slow-delta (0.1–4.0 Hz) and an increase in the presence of fast-gamma (32.1–128.0 Hz) frequency ranges of the cerebellum. An increase in gamma frequency persists for 120 min after. Moreover, two weeks later, the spectral features of cerebellar activity still showed a high prevalence of gamma frequency activity (20%), indicating that cerebral injury could induce chronic changes in cerebellar electrical activity [22,26]. These results suggest that spectral changes in the gamma frequency band may indicate the cerebellar state after acute or chronic cerebral injury. Gamma-frequency band oscillations may also have a protective role in the cerebellum, helping stabilize postsynaptic transmission after traumatic brain injury [162]. Cerebellar lesions can affect the oscillatory dynamics of the delta frequency band and disrupt the precise temporal coding of sensory information [85]. Further research is needed to better understand the mechanisms underlying cerebellar trauma pathogenesis, and experimental models could be an important step toward that objective.

## 7. Cerebellum and Neurodegenerative Diseases

Alzheimer’s disease (AD) is a degenerative brain disorder of middle or late life that destroys neurons and connections in the cerebral cortex, leading to significant loss of brain mass and cognitive impairment [163]. The core pathogenesis of AD involves amyloid-β (Aβ) deposition, hyperphosphorylated tau (p-Tau), neuroinflammation, and oxidative stress [164,165,166,167]. The deposition of the amyloid β protein (Aβ) is a histopathologic hallmark of AD. There is a hierarchical involvement of brain regions in the expansion of Aβ pathology. In the first of five phases in the evolution of β-amyloidosis, Aβ deposits are found exclusively in the neocortex. The second phase is characterized by the additional involvement of allocortical brain regions. In phase 3, diencephalic nuclei, the striatum, and the cholinergic nuclei of the basal forebrain exhibit Aβ deposits as well. Several brainstem nuclei become additionally involved in phases 4 and 5, which are characterized by cerebellar Aβ-deposition [168].

Recent research highlights the cerebellum’s involvement in the disease’s pathogenesis. Amyloid plaques were found in the cerebellar cortex in cases of senile dementia of the Alzheimer type, and the majority occurred in the molecular layer [169]. The highest incidence of amyloid plaques in the cerebellum was found in the group of patients who developed dementia before 65 years of age (93%) [170]. Alzheimer’s disease is associated with significant anatomical tissue atrophy in the Crus I of the cerebellum [171]. The loss of cerebellar white matter occurs with aging and is greater than the loss of gray matter [172]. Alzheimer’s disease patients had significantly fewer Purkinje cells in the vermis than healthy subjects [173]. Patients with Alzheimer’s disease showed Purkinje cell loss and a marked decrease in dendritic arborization and spine density [174,175]. Individuals with Alzheimer’s disease showed poorer memory performance and an increase in fMRI signals in the frontal and temporal lobes, suggesting underlying pathological processes [176]. In Alzheimer’s disease patients, correlations between cerebellar fMRI activity and memory accuracy were found, suggesting involvement of the cerebellar network in memory performance [177]. Posterior cerebellar lobes were significantly smaller in AD patients when compared to healthy controls. In the AD group, atrophy of the posterior cerebellar regions was associated with poorer cognitive performance [178]. Alzheimer’s disease patients exhibited gait dysfunction associated with cerebellar anterior lobe atrophy [179].

An increase in Purkinje cell and deep cerebellar nuclear neuron firing was established in a mouse model of Alzheimer’s [180]. fMRI showed that Alzheimer’s dementia is associated with the presence of a low-frequency band (0.01–0.027 Hz) in the cerebellum, and extensive electrophysiological literature has shown a pathological ‘slowing’ of neuronal activity in patients on the Alzheimer’s disease spectrum [181]. Furthermore, decreased beta-frequency (16–24 Hz) oscillatory activity was observed in MEG recordings from the cerebella of patients with cognitive dysfunction [182]. The slow frequencies—delta (0.1–4.0 Hz) and theta (4.1–8.0 Hz)—were increased, while higher frequencies—alpha (8.1–15.0 Hz), beta (15.1–32.0 Hz), and gamma (32.1–128.0 Hz)—were decreased in the mean power spectra of cerebellar local field potentials in an animal model of Alzheimer’s disease [23,24,183,184]. Increases in low-frequency and decreases in high-frequency neural oscillations are used as EEG clinical markers of the onset and possibly progression of AD. Moreover, regional amyloid β accumulation could predict the magnitude of this pathological neural slowing effect [185]. Older subjects showed greater electromagnetic activity originating from the left crus II and lobule VIIb of the cerebellum, and these regions may be essential for the earliest possible detection of AD risk, enabling early intervention before symptom onset [186].

Parkinson’s disease is a progressive neurodegenerative disease that causes the death of neurons in the brain responsible for the production of dopamine, a neurotransmitter key to movement control [187]. Locomotion is a complex process that involves automatic, emotional, and volitional control [188]. In non-automatic gait, the initiation command is generated in the cerebral cortex and executed by the thalamocortical, corticobulbar, and spinal projection networks [189]. Postural, balance, and movement control are regulated by the brainstem and spinal cord after gait is volitionally initiated. Meanwhile, the cerebellum simultaneously receives sensory feedback from the spinal cord and feed-forward information from the cortex to fine-tune movements [5].

Tremor is a hyperkinetic movement associated with cerebellar dysfunction. Essential tremor is characterized by tremor during action, while Parkinsonian tremor is associated with rest. Striato-thalamo-cortical motor circuit dysfunction has been implicated clearly in Parkinson’s disease (PD). The cerebellum is also an important component of motor control and influences cortical activity via cerebello-thalamo-cortical (CTC) circuits [41]. Increased recruitment of the CTC motor circuit with PD progression suggests its role in accommodation to, or the pathophysiology of, PD [190].

EEG could be very useful for characterizing alterations in neurophysiological oscillatory activity associated with Parkinson’s disease and for monitoring disease progression [191]. Findings suggest a slowing of cortical activity in patients with PD, reflected in increased slow-band wave activity and decreased fast-band wave activity at rest and during complex movement execution, mainly in the central and frontal cortices [86]. In PD a main frequency of motor cortex activity corresponds to double the tremor frequency [192]. PD patients showed attenuation of mid-cerebellar theta-band (4–7 Hz) activity during cognitive and motor tasks. Theta power (4–7 Hz) was also profoundly lower in mid-cerebellar regions during postural control [193]. Abnormally high-frequency bursting activity was observed in neurons of the deep cerebellar nuclei in rapid-onset dystonia-Parkinsonism [194].

Tremor can be effectively suppressed by deep brain stimulation of the thalamus when receiving cerebellar outflow, providing convincing evidence of cerebellar involvement in tremor disorders [195]. Stimulation of the cerebellum and motor cortex decreases dyskinesia in Parkinson’s disease [196]. Levodopa, which is used to treat the symptoms of Parkinson’s disease, increases functional connectivity in the cerebellum and its connections with other motor-related brain areas, such as the basal ganglia and thalamus, and improves motor symptoms [197,198].

## 8. Cerebellum and Neuropsychiatric Disorders

Human electrophysiological data supporting cerebellar involvement in cognitive and emotional functions are lacking [199]. Single-pulse transcranial magnetic stimulation over the cerebellar vermis increases EEG theta activity in healthy humans. Theta activity is associated with the septo-hippocampal complex, an important brain structure involved in cognition and emotion. These findings demonstrate cerebellar involvement in modulating the core frequencies underlying cognitive and emotive aspects of human behavior [200]. Schizophrenia is characterized by psychotic symptoms and cognitive impairment [201]. Approximately half of the cerebellar cortex is associated with higher-level cognitive and affective functions [202]. Vermal abnormalities are more frequently noted in tasks that use limbic regions (responsible for emotion), while more lateral neocerebellar regions are abnormal in tasks that use neocortical regions (responsible for memory encoding and retrieval) [203]. Cerebellar gray matter volume was reduced in schizophrenia relative to healthy controls [204]. It was reported that Purkinje cell density decreased, or that Purkinje cell size decreased [205]. The latter finding is particularly significant, since Purkinje cells play a key role in modulating the cerebellum’s output to the cerebral cortex by providing input to the “deep nuclei”, such as the dentate nucleus. The deep nuclei in turn provide the sole output from the cerebellum to the cerebral cortex. Dysfunctional neural circuitry is involved in abnormalities of perception and cognition in patients with schizophrenia.

The prefrontal–thalamic–cerebellar network is activated when healthy subjects recall complex narrative material, but is dysfunctional in schizophrenic patients when they perform the same task. These results support a role for the cerebellum in cognitive functions and suggest that patients with schizophrenia may suffer from a “cognitive dysmetria” due to dysfunctional prefrontal–thalamic–cerebellar circuitry [206]. Gamma oscillations are essential for integrating information within neural circuits and have therefore been associated with many perceptual and cognitive processes in healthy human subjects and animals. The gamma frequency band is increased in schizophrenia. The GABAergic interneuronal system is thought to be responsible for gamma-range deficits in schizophrenia [87,207]. Cerebellar repetitive transcranial magnetic stimulation (rTMS) reduces gamma oscillatory activity of the cerebral cortex in patients suffering from schizophrenia. Cerebellar-rTMS might be an effective adjunct to treat intricate and lingering negative and affective symptoms of schizophrenia [87]. The beta frequency band is increased in schizophrenia [88]. Low-frequency brain oscillations are reduced in early-stage schizophrenia and may provide evidence for early diagnosis of the disease [208]. Besides the motor domain, the cerebellum is involved in affect regulation [209]. Multidisciplinary evidence has demonstrated topographic organization of the cerebellum. The anterior lobe and lobule VIII contain the representation of the sensorimotor cerebellum. Lobules VI and VII include representations of the cognitive cerebellum, while the posterior vermis is an anatomical substructure of the limbic cerebellum [210]. Increased volume of lobule IX has been demonstrated in patients with major depression [211].

Depression is characterized by slowing of EEG and increased theta and alpha power. Reduced levels of theta and alpha rhythms in EEG can be good indicators in the treatment of major depression [89]. Data have also shown that gamma rhythms can be decreased or increased in major depressive disorder [90]. Recent studies implicate involvement of the cerebellum in anxiety [212]. Delta–beta cross-frequency correlation is a widely used measure in the investigation of social anxiety. Data showed a stronger positive correlation between delta and beta frequencies in patients suffering from anxiety [91]. Autism spectrum disorder (ASD) is characterized by social and communication impairments, and by restricted and stereotyped behaviors [213]. Individuals with ASD had less absolute delta than controls [92]. It was found that levels of GABAB receptor 1 were significantly decreased in Brodmann’s areas 9 and 40, and in the cerebellum, while GABAB receptor 2 was significantly reduced only in the cerebellum [214].

## 9. Use of ECeG in Identifying Common Mechanisms Underlying Cerebellar Dysfunction

Given the cerebellar involvement in the pathogenesis of a wide range of neurological conditions, an important question arises: Are there any general models that account for its failure to perform and to regulate neurophysiological processes such as motor control, cognition, and affective behavior? Is ECeG sensitive enough to capture some of these mechanisms? These mechanisms are still underexplored, and the open questions they raise deserve full scientific attention. In neurophysiology, there has been a long-standing search to anatomically locate the so-called “internal forward models”, or neural mechanisms that can mimic the input–output or output–input properties of the motor apparatus and external objects, predicting sensory consequences from efference copies of motor commands in sensorimotor integration and in higher cognitive function. This model is designed and refined to predict motor outcomes by comparing them with sensory feedback, which, in turn, generates prediction errors that improve prediction accuracy [215]. As supported by fMRI grip force–load force coupling studies, some suggest that these mechanisms, which accurately represent the input–output properties of the motor apparatus, are located in the cerebellum [216,217]. Others, such as Mangalam [218], are more critical of this “metaphorical” computational framework, moving beyond the internal model paradigm. Mangalam [218] convincingly argues that sensorimotor neuroscience can develop more robust explanatory frameworks to capture the emergent, context-sensitive properties of biological movement without relying on physiologically complex computational metaphors.

In any case, there may be common mechanisms underlying failure of the cerebellum’s internal forward model to deliver sensorimotor and cognitive integration, manifesting as cerebellar syndrome, which is categorized into cerebellar motor syndrome (CMS), vestibulocerebellar syndrome (VCS), and cerebellar cognitive affective syndrome (CCAS), or Schmahmann syndrome (SS) [219]. The first two types are characterized by impaired motor control and coordination, imbalance, abnormal extremity and eye movements, dysarthria, and gait disorders [219,220,221,222]. In contrast, the third is characterized by a broader spectrum of cognitive and affective symptoms, including impairments in executive function, spatial cognition, language processing, and emotional regulation [219].

The first major question here is which external or internal events trigger neuropathological processes in the cerebellum. Some recent studies provide indications in both directions. For example, Smith et al. [223] recently implicated biallelic POLG variants and pathogenic mitochondrial DNA variants involved in pediatric and adult mitochondrial disease (m.3243A > G, m.8344A > G, m.13094T > C, and m.14709T > C) in the loss of inhibitory Purkinje cells, probably due to oxidative phosphorylation protein deficiencies, which the authors claim to be more severe than in the predominantly excitatory neuronal populations of the granule cell layer and dentate nucleus. This clearly suggests that, in some cerebellar pathologies, internal genetic factors play an important role in cerebellar dysfunction. Genetic studies in general have revealed that some types of ataxias are autosomal dominant, while others are autosomal recessive [224].

In contrast, many external and environmental factors, such as alcohol consumption, deficiencies of nutrients such as vitamins B1 and B12, traumatic head injuries, viral and bacterial infections, and immune-mediated causes, can lead to cerebellar dysfunction [224,225]. The second major question concerning integrated mechanisms that may connect various cerebellar functional roles and their dysfunction across pathological states is the potential cerebellar-specific or nonspecific mechanisms underlying cerebellar (dys)function. Recently, it was reported that prenatal exposure to valproic acid (VPA) in a mouse model of ASD led to cerebellar impairment associated with cognitive impairment in ASD through dysfunction of glutamate receptor signaling and disruptions in axon-guidance signaling [226,227]. More specifically, it appears that a gene called GRIN2B, which encodes the Grin2b protein together with other NMDA receptor subunits, is critical for establishing neuronal communication, which is essential for learning, memory, and synaptic plasticity. By linking synaptic neurotransmission and neurodevelopmental disorders, this molecule is implicated in a rare neurodevelopmental disorder called GRIN2B-related neurodevelopmental disorder, which manifests as intellectual disability, developmental delay, motor impairments, autism spectrum disorder, and possibly epilepsy [226,227]. Thus, Grin2b, along with other molecules and pathways, may represent a molecular target and a link connecting these neurological conditions in the cerebellum and beyond. In the context of autism and related disorders characterized by pronounced reductions in social skills, the cerebellum appears to play an important role in the wiring events underlying higher cognitive functions, including social behavior and language [228].

Convergently, all these changes at the molecular and cellular level more broadly encompass what Manto and Marmolino [229] (p. 417) classified as impairment of DNA repair and replication, deregulation of transcription/deficits of processing/transport of RNA, abnormal protein transport and misfolding, aggregates at both the nuclear and cytosolic level, activation of caspases, apoptosis, involvement of autophagic mechanisms, oxidative stress and mitochondrial dysfunction, excitotoxicity, abnormal lipid metabolism, impaired axonal transport and vesicle trafficking, and defects of neurotransmission. We still do not have a complete picture of the combined or separate molecular mechanisms triggered by external and/or internal influences that cause neurodegeneration in the cerebellum and how this process affects the cerebro-cerebellar system, a network of bidirectional loops between the cerebellum and cerebral cortex crucial for motor control, cognition, and emotion [230].

The question posed at the beginning of this section regarding the role of ECeG in elucidating the mechanisms of cerebellar function and dysfunction remains only partially answered and merits further scientific exploration. The discussion of the advantages and limitations of EEG, including the ECeG technique, has been ongoing since the 1920s and Berger’s pioneering work in EEG methodology [231]. More specifically, the evaluation of the prospects and limitations of ECoG (electrocorticography or intracranial electroencephalography [iEEG]), stereotactic EEG (depth electrodes), and ECeG techniques, along with the underlying methodologies for their implementation in research, is informed by their use in animal studies [232]. Across the wealth of these studies, two major orientations regarding their use appear: (1) evaluating their similarities and differences with noninvasive human EEG to verify the existing use of scalp EEG for the diagnosis and monitoring of the onset and progression of neurological diseases; and (2) their use in fundamental research as additional strategies to complement molecular and cellular studies of cerebro-cerebellar pathologies in small laboratory animals. Both orientations converge on one conclusion: EEG and its derivatives still play an important role in research and clinical practice and, when possible, should be complemented with fMRI in clinical settings and, in animal research, with molecular and cellular data to support sound research and clinical practice [233,234]. Indeed, Parvizi and Kastner [235] hold that iEEG can reveal anatomically precise information about functional interactions governed by molecular processes within and across neural networks during different stages of neural computation, beyond simply replicating what is already known from noninvasive recordings in humans and invasive recordings in animals.

The precision of iEEG allows researchers to selectively trace the temporal dynamics of neuronal populations at the millisecond time scale and the millimeter spatial scale. We believe this applies to ECoG without reservation. Nevertheless, intraoperative sensorimotor electrocorticography (ECoG) has been reported to be a superior technique for accurate grip-force decoding compared with subthalamic LFP [236]. Some cortical sources are not visible on scalp EEG but become visible when electrodes are placed intracortically or at specific depths via stereotaxic implantation [234]. This realization has enabled the combined use of scalp EEG and ECoG since the 1950s; this method continues to be used today and has been used prospectively in 21st-century cognitive, clinical, and computational neuroscience [234].

However, EEG and its various modifications lack the spatial resolution to capture anatomical locations relevant to certain functional tasks, whether on the surface of the cerebrum or cerebellum, or deep within their layers [237]. That is why many methods of functional neuroimaging, such as fMRI, which use magnetic fields and radio waves to produce detailed images of specific regions, entire brain structures, and the whole brain, have been developed in recent decades [237,238]. Given the highly praised potential of fMRI in the spatial domain, the following question arises: What are the advantages of EEG and ECeG over fMRI? The answer is straightforward. The spatial and temporal profiles of these two techniques differ significantly, as do the sources of the signals measured by fMRI and EEG [239,240]. For example, EEG has millisecond resolution, which is much higher than fMRI’s temporal resolution, characterized by a lag of 6–7 s in the hemodynamic response [239,240].

In contrast, fMRI has superior spatial resolution, encompassing whole-brain structures, compared to EEG electrodes, which collect electrical signals from a single location and a limited number of neighboring neurons. For this and many other reasons, over the past few decades numerous studies have attempted to combine EEG and fMRI in human and animal research and to establish a mathematical model of the so-called coupling between neuronal activity; cerebral (cerebellar) metabolic rates of glucose and oxygen consumption; cerebral (cerebellar) blood flow (CBF); electroencephalography (EEG); and blood oxygenation level-dependent (BOLD) responses [241]. There are, of course, many technical, medical, and research papers detailing EEG and fMRI and their combined use in the study of the cerebellum and other brain structures, which we will not discuss further here. To achieve the most credible and verifiable results in cerebellum research, it is essential to complement these methods with additional neuroimaging techniques, such as fMRI, and to incorporate molecular and cellular findings. This comprehensive approach will ensure the highest standards in research practices and perhaps lead to the next revolution in cerebellar neurophysiology.

## 10. Conclusions

Given the cerebellum’s crucial role in motor control, auditory perception, vocalization, speech, memory, emotional control, and social cognition, this review highlights the importance of studying it using the ECeG technique across various morphofunctional brain disorders. Despite certain limitations, ECeG has proven practical, valuable, and reliable for detecting and predicting the potential consequences of cerebellar functional changes in animals. Very little is known about the rhythmic activity of the cerebellum in health and disease. Nevertheless, all the studies discussed here, including ours, expand the horizons of cerebellar modulation of brain rhythms. New brain imaging techniques, such as MRI alone and combined with EEG/ECG, provide a more dynamic picture of brain activity and its role in adaptability during wakefulness and sleep in animals and humans, both in health and disease. In this context, the cerebellum’s role in global brain dynamics through segregation and integration, as fundamental processes of brain organization, will become clearer through further-optimized EEG/ECeG research approaches, including multi-electrode-array EEG technology. Many studies, including ours, indicate that these goals can only be achieved by combining linear and nonlinear methods with advanced statistical techniques to extract detailed information from cerebellar ECeG and other electrophysiological time series. In animal models, establishing functional connections between the cerebellum and the rest of the brain can deepen our understanding of the brain’s context-dependent responses and inform translational research aimed at deciphering human brain pathologies. In this regard, further studies are needed to shed more light on the context-dependent dynamical oscillatory activity of the cerebellum.

## Figures and Tables

**Table 1:** Cerebellar oscillatory activity during sensorimotor integration and sleep.

Physiological State	Main Findings	Species	Ref.
**Sensory-motor** **integration**	During immobility and temporal encoding of sensory data, the dominant rhythm is theta at 7–8 Hz.	Rat	[29]
	Synchronization, rhythmicity, and learning in the cerebellum are characterized by a 3–12 Hz. rhythm.	Rat	[30]
	In awake animals, during periods of immobility and readiness to respond to an auditory stimulus, a rhythm dominated by 13–18 Hz is observed.	Monkey	[31]
	When the animals quietly awaited a reward after a stimulus appeared, or moved in response to a stimulus, their anticipation of events was characterized by frequencies between 13 and 25 Hz.	Monkey	[32]
	Spatiotemporal coding between the cortex and cerebellum occurs at 30–80 Hz and 80–160Hz.		[33]
	Precise timing, which is essential for controlling complex, coordinated, rapid movements, occurs at 200 Hz.		[34]
**Sleep**	Circadian oscillations in the firing rate of Purkinje cells.		[35]
	Purkinje cells project to the medial parabrachial nucleus, which is important in sleep regulation.	Mouse	[36]
	Purkinje cells exhibited increased firing activity prior to the transition from sleep to wakefulness.	Mouse	[37]

**Table 2:** The cerebellum’s vital role across neurological states and disorders.

Pathological Condition	Main Findings	Species	Ref.
**Anesthesia**	Anesthetics disrupt the delicate balance of neuronal communication in the cerebellum, affecting processes such as sleep-wake cycles and cognitive functions.	Human	[76,77]
	The cerebellum participates in conscious recovery from anesthesia.	Human	[78]
	Anesthetics cause prominent slow delta and theta frequency oscillations.	Rats	[25]
**Epilepsy**	Cerebellar impairments have been observed in patients with epilepsy.	Human	[79]
	Cerebellar degeneration was most prevalent in patients with temporal lobe epilepsy.	Human	[80]
	Seizures arise from the cerebral cortex, glossing over infratentorial structures such as the cerebellum that are believed to modulate rather than generate seizures.	Human	[81]
	Magnetoencephalography showed paroxysmal activity at the cerebellar vermis and hemispheres in patients with epilepsy.	Human	[82]
	It was shown that the cerebellum showed contralateral activation 14 s after ictal onset in the motor cortex.	Human	[83]
**Traumatic Brain** **Injury**	Generally, reduced spontaneous cerebellar electric activity was present in acute traumatic brain injury patients.	Human	[84]
	Cerebellar lesions can affect the oscillatory dynamics of the delta frequency band and disrupt the precise temporal coding of sensory information.	Human	[85]
	Spectral changes in the gamma frequency band may indicate the cerebellar state after acute or chronic cerebral injury.	Rats	[26]
**Neurodegenerative disease**	Increases in low-frequency and decreases in high-frequency neural oscillations could be a stable biomarker with potential clinical utility for Alzheimer’s disease.	Rats	[23]
	Patients with PD show increased slow-band wave activity and decreased fast-band wave activity.	Human	[86]
**Neuropsychiatric** **disorders**	Gamma frequency band is increased in schizophrenia.	Human	[87]
	Beta frequency band is increased in schizophrenia.	Human	[88]
	Depression is characterized by a slowing of the EEG and increased level of theta and alpha bands.	Human	[89]
	Gamma rhythms can be decreased or increased in major depressive disorder.	Human	[90]
	Data showed a stronger positive correlation between delta and beta frequencies in patients suffering from anxiety.	Human	[91]
	Individuals with ASD had less absolute delta power than controls.	Human	[92]

## Data Availability

No new data were created or analyzed in this study. Data sharing is not applicable to this article.

## References

[1] Carey M.R. (2024). The cerebellum. Curr. Biol..

[2] Koziol L.F., Budding D., Andreasen N., Arrigo S., Bulgheroni S., Imamizu H., Ito M., Manto M., Marvel C., Parker C. (2014). Consensus paper: The cerebellum’s role in movement and cognition. Cerebellum.

[3] Rudolph S., Badura A., Lutzu S., Pathak S.S., Thieme A., Verpeut J.L., Wagner M.J., Yang M.J., Yang Y.-M., Fioravante D. (2023). Cognitive-affective functions of the cerebellum. J. Neurosci..

[4] Sivalingam A.M., Pandian A. (2024). Cerebellar roles in motor and social functions and implications for ASD. Cerebellum.

[5] Manto M., Bower J.M., Conforto A.B., Delgado-García J.M., da Guarda S.N., Gerwig M., Habas C., Hagura N., Ivry R.B., Mariën P. (2012). Consensus paper: Roles of the cerebellum in motor control-the diversity of ideas on cerebellar involvement in movement. Cerebellum.

[6] Faris P., Pischedda D., Palesi F., D’Angelo E. (2024). New clues for the role of cerebellum in schizophrenia and the associated cognitive impairment. Front. Cell. Neurosci..

[7] Phillips J.R., Hewedi D.H., Eissa A.M., Moustafa A.A. (2015). The cerebellum and psychiatric disorders. Front. Public Health.

[8] Voogd J., Glickstein M. (1998). The anatomy of the cerebellum. Trends Cogn. Sci..

[9] Heuer K., Traut N., de Sousa A.A., Valk S.L., Clavel J., Toro R. (2023). Diversity and evolution of cerebellar folding in mammals. eLife.

[10] Kandel E.R., Schwartz J.H., Jessell T.M. (2000). Principles of Neural Science.

[11] Llinas R.R., Walton K.D., Lang E.J., Shepherd G.M. (2004). Cerebellum. The Synaptic Organization of the Brain.

[12] Prati J.M., Pontes-Silva A., Lepesteur Gianlorenco A.K. (2024). The cerebellum and its connections to other brain structures involved in motor and non-motor functions: A comprehensive review. Behav. Brain. Res..

[13] Niedermeyer E., da Silva F.L. (2005). Electroencephalography: Basic Principles, Clinical Applications, and Related Fields.

[14] Chow P.K.H., Ng R.T.H., Ogden B.E. (2008). Using Animal Models in Biomedical Research.

[15] Adrian E.D. (1935). Discharge frequencies in the cerebral and cerebellar cortex. J. Physiol. Lond..

[16] Dow R.S. (1938). The electrical activity of the cerebellum and its functional significance. J. Physiol..

[17] Eccles J.C. (1967). Circuits in the cerebellar control of movement. Proc. Natl. Acad. Sci. USA.

[18] Marr D.A. (1969). Theory of cerebellar cortex. J. Physiol..

[19] Albus J.S. (1971). A theory of cerebellar function. Math. Biosci..

[20] Llinás R., Sugimori M. (1980). Electrophysiological properties of in vitro Purkinje cell dendrites in mammalian cerebellar slices. J. Physiol..

[21] Ito M. (2000). Mechanisms of motor learning in the cerebellum. Brain Res..

[22] Ćulić M., Martać Blanuša L., Grbić G., Spasić S., Janković B., Kalauzi A. (2005). Spectral analysis of cerebellar activity after acute brain injury in anesthetized rats. Acta Neurobiol. Exp. (Wars).

[23] Ćulić M., Martać L., Grbić G., Kesić S., Spasić S., Sekulić S., Lalošević D., Čapo I., Gantchev V. (2007). Aluminum Toxicity in Rat Brain: Electrophysiological, Histological and Behavioral Evidence.

[24] Keković G., Ćulić M., Martać L., Stojadinović G., Čapo I., Lalošević S., Sekulić S. (2010). Fractal dimension values of cerebral and cerebellar activity in rats loaded with aluminum. Med. Biol. Eng. Comput..

[25] Keković G., Stojadinović G., Martać L., Podgorac J., Sekulić S., Ćulić M. (2010). Spectral and fractal measures of cerebellar and cerebral activity in various types of anesthesia. Acta Neurobiol. Exp. (Wars).

[26] Spasić S., Ćulić M., Grbić G., Martać L., Sekulić S., Mutavdzić D. (2008). Spectral and fractal analysis of cerebellar activity after single and repeated brain injury. Bull. Math. Biol..

[27] Stojadinović G., Martać L., Podgorac J., Spasić S., Sekulić S., Kesić S. (2020). The effects of Nembutal on the intracerebellar EEG activity revealed by spectral and fractal analysis. Arch. Biol. Sci..

[28] Podgorac-Kojadinović J., Petković B., Kalauzi A., Martać L., Sekulić S., Stojadinović G. (2025). Characterization of cerebellar electrocortical dynamics under ether anesthesia in rats: A preliminary study using linear spectral and nonlinear fractal analyses. Arch. Biol. Sci..

[29] Hartmann M.J., Bower J.M. (1988). Oscillatory activity in the cerebellar hemispheres of unrestrained rats. J. Neurophysiol..

[30] D’Angelo E., Nieus T., Maffei A., Armano S., Rossi P., Taglietti V., Fontana A., Naldi G. (2001). Theta-frequency bursting and resonance in cerebellar granule cells: Experimental evidence and modeling of a slow K+-dependent mechanism. J. Neurosci..

[31] Pellerin J.P., Lamarre Y. (1997). Local field potential oscillations in primate cerebellar cortex during voluntary movement. J. Neurophysiol..

[32] Courtemanche R., Pellerin J.P., Lamarre Y. (2002). Local field potential oscillations in primate cerebellar cortex: Modulation during active and passive expectancy. J. Neurophysiol..

[33] Middleton S.J., Racca C., Cunningham M.O., Traub R.D., Monyer H., Knöpfel T., Schofield I.S., Jenkins A., Whittington M.A. (2008). High-frequency network oscillations in cerebellar cortex. Neuron.

[34] de Solages C., Szapiro G., Brunel N., Hakim V., Isope P., Buisseret P., Rousseau C., Barbour B., Léna C. (2008). High-frequency organization and synchrony of activity in the Purkinje cell layer of the cerebellum. Neuron.

[35] Mordel J., Karnas D., Pévet P., Isope P., Challet E., Meissl H. (2013). The Output Signal of Purkinje Cells of the Cerebellum and Circadian Rhythmicity. PLoS ONE.

[36] Hashimoto M., Yamanaka A., Kato S., Tanifuji M., Kobayashi K., Yaginuma H. (2018). Anatomical evidence for a direct projection from Purkinje cells in the mouse cerebellar vermis to medial parabrachial nucleus. Front. Neural Circuits.

[37] Zhang L.-B., Zhang J., Sun M.-J., Chen H., Yan J., Luo F.-L., Yao Z.-X., Wu Y.-M., Hu B. (2020). Neuronal activity in the cerebellum during the sleep-wakefulness transition in mice. Neurosci. Bull..

[38] Waterhouse B.D., Predale H.K., Plummer N.W., Jensen P., Chandler D.J. (2022). Probing the structure and function of locus coeruleus projections to CNS motor centers. Front. Neural Circuits.

[39] Davie J.T., Clark B.A., Häusser M. (2008). The origin of the complex spike in cerebellar Purkinje cells. J. Neurosci..

[40] McKay B.E., Engbers J.D., Mehaffey W.H., Gordon G.R.J., Molineux M.L., Bains J.S., Turner R.W. (2007). Climbing fiber discharge regulates cerebellar functions by controlling the intrinsic characteristics of purkinje cell output. J. Neurophysiol..

[41] Afifi A.K., Bergman R.A. (1998). Functional Neuroanatomy: Text and Atlas.

[42] Cousins J.N., Fernández G. (2019). The impact of sleep deprivation on declarative memory. Prog. Brain Res..

[43] Maquet P., Schwartz S., Passingham R., Frith C. (2003). Sleep-related consolidation of a visuomotor skill: Brain mechanisms as assessed by functional magnetic resonance imaging. J. Neurosci..

[44] Stenson A.R., Whitney P., Hinson J.M. (2023). Effects of total sleep deprivation on components of top-down attentional control using a flexible attentional control task. J. Sleep Res..

[45] Schimke E.A.E., Angwin A.J., Cheng B.B.Y., Copland D.A. (2021). The effect of sleep on novel word learning in healthy adults: A systematic review and meta-analysis. Psychon. Bull. Rev..

[46] Mantua J., Bessey A.F., Mickelson C.A., Choynowski J.J., Noble J.J., Burke T.M., McKeon A.B., Sowden W.J. (2021). Sleep and high-risk behavior in military service members: A mega-analysis of four diverse U.S. Army units. Sleep.

[47] Beaty R.E., Silvia P.J., Nusbaum E.C., Vartanian O. (2013). Tired minds, tired ideas? Exploring insomnia and creativity. Think. Ski. Creat..

[48] Krueger J.M., Frank M.G., Wisor J.P., Roy S. (2016). Sleep function: Toward elucidating an enigma. Sleep Med. Rev..

[49] McCarley R.W. (2007). Neurobiology of REM and NREM sleep. Sleep Med..

[50] Moser D., Anderer P., Gruber G. (2009). Sleep classification according to AASM and Rechtschaffen & Kales: Effects on sleep scoring parameters. Sleep.

[51] Amzica F., Steriade M. (1998). Electrophysiological correlates of sleep delta waves. Electroencephalogr. Clin. Neurophysiol..

[52] Blumberg M.S., Lesku J.A., Libourel P.-A. (2020). What is REM sleep?. Curr. Biol..

[53] Buzsáki G. (2002). Theta oscillations in the hippocampus. Neuron.

[54] Llinas R., Ribary U. (1993). Coherent 40-Hz oscillation characterizes dream state in humans. Proc. Natl. Acad. Sci. USA.

[55] Rath M.F., Rovsing L., Møller M. (2014). Circadian oscillators in the mouse brain: Molecular clock components in the neocortex and cerebellar cortex. Cell Tissue Res..

[56] Anaclet C., Lin J.-S., Vetrivelan R., Krenzer M., Vong L., Fuller P.M., Lu J. (2012). Identification and characterization of a sleep-active cell group in the rostral medullary brainstem. J. Neurosci..

[57] Xu W., De Carvalho F., Clarke A.K., Jackson A. (2021). Communication from the cerebellum to the neocortex during sleep spindles. Prog. Neurobiol..

[58] Canto C.B., Onuki Y., Bruinsma B., van der Werf Y.D., De Zeeuw C.I. (2017). The sleeping cerebellum. Trends Neurosci..

[59] Dang-Vu T.T., Schabus M., Desseilles M. (2008). Spontaneous neural activity during human slow wave sleep. Proc. Natl. Acad. Sci. USA.

[60] Jahnke K., von Wegner F., Morzelewski A., Borisov S., Maischein M., Steinmetz H., Laufs H. (2012). To wake or not to wake? The two-sided nature of the human K-complex. Neuroimage.

[61] Schabus M., Dang-Vu T.T., Albouy G., Maquet P. (2007). Hemodynamic cerebral correlates of sleep spindles during human non-rapid eye movement sleep. Proc. Natl. Acad. Sci. USA.

[62] Dang D., Cunnington D. (2010). Excessive daytime somnolence in spinocerebellar ataxia type 1. J. Neurol. Sci..

[63] Hong C.C.-H., Harris J.C., Pearlson G.D. (2009). FMRI evidence for multisensory recruitment associated with rapid eye movements during sleep. Hum. Brain Mapp..

[64] Marchesi G.F., Strata P. (1971). Mossy and climbing fiber activity during phasic and tonic phenomena of sleep. Pflug. Arch..

[65] Barmack N.H., Yakhnitsa V. (2011). Topsy turvy: Functions of climbing and mossy fibers in the vestibulo-cerebellum. Neuroscientist.

[66] Brown S.T., Medina-Pizarro M., Holla M., Vaaga C.E., Raman I.M. (2024). Simple spike patterns and synaptic mechanisms encoding sensory and motor signals in Purkinje cells and the cerebellar nuclei. Neuron.

[67] Mano N. (1970). Changes of simple and complex spike activity of cerebellar Purkinje cells with sleep and waking. Science.

[68] McCarley R.W., Hobson J.A. (1972). Simple spike firing patterns of cat cerebellar Purkinje cells in sleep and waking. Electroencephalogr. Clin. Neurophysiol..

[69] Palmer C. (1979). Interpositus and fastigial unit activity during sleep and waking in the cat. Electroencephalogr. Clin. Neurophysiol..

[70] Torres-Herraez A., Watson T.C., Rondi-Reig L. (2022). Delta oscillations coordinate intracerebellar and cerebello-hippocampal network dynamics during sleep. J. Neurosci..

[71] Cunchillos J.D., De Andres I. (1982). Participation of the cerebellum in the regulation of the sleep-wakefulness cycle. Results in cerebellectomized cats. Electroencephalogr. Clin. Neurophysiol..

[72] Arnulf I., Bejjani B., Garma L., Bonnet A.M., Houeto J.L., Damier P., Derenne J.P., Agid Y. (2000). Improvement of sleep architecture in PD with subthalamic nucleus stimulation. Neurology.

[73] Prinz P.N., Peskind E.R., Vitalino P.P., Raskind M.A., Eisdorfer C., Zemcuznikov N., Gerber C.J. (1982). Changes in the sleep and waking EEGs of nondemented and demented elderly subjects. J. Am. Geriatr. Soc..

[74] Sonni A., Kurdziel L., Baran B., Spencer R. (2014). The effects of sleep dysfunction on cognition, affect, and quality of life in individuals with cerebellar ataxia. J. Clin. Sleep Med..

[75] Song B., Zhu J.-C. (2021). A narrative review of cerebellar malfunctions and sleep disturbances. Front. Neurosci..

[76] Kain Z.N., Caldwell-Andrews A.A. (2003). Sleeping characteristics of adults undergoing outpatient elective surgery: A cohort study. J. Clin. Anesth..

[77] Patel D., Lunn A.D., Smith A.D., Lehmann D.J., Dorrington K.L. (2016). Cognitive decline in the elderly after surgery and anesthesia: Results from the Oxford Project to Investigate Memory and Aging (OPTIMA) cohort. Anaesthesia.

[78] Zhu J., Chen C., Liu X., He M., Fang Y., Wang L., Jia J., Guo J., Zhao Z., Gao C. (2024). Cerebellar Purkinje cell firing promotes conscious recovery from anesthesia state through coordinating neuronal communications with motor cortex. Theranostics.

[79] Streng M.L., Krook-Magnuson E. (2021). The cerebellum and epilepsy. Epilepsy Behav..

[80] Ibdali M., Hadjivassiliou M., Grünewald R.A., Shanmugarajah P.D. (2021). Cerebellar degeneration in epilepsy: A systematic review. Int. J. Environ. Res. Public Health.

[81] Marcián V., Filip P., Bareš M., Brázdil M. (2016). Cerebellar dysfunction and ataxia in patients with epilepsy: Coincidence, consequence, or cause?. Tremor Other Hyperkinet Mov. (NY).

[82] Kotini A., Mavraki E., Anninos P., Piperidou H., Prassopoulos P. (2010). Magnetoencephalographic findings in two cases of juvenile myoclonus epilepsy. Brain Topogr..

[83] Mohamed I.S., Otsubo H., Ferrari P., Ochi A., Snead O.C., Douglas C. (2011). Neuromagnetic cerebellar activation during seizures arising from the motor cortex. Epilepsy Res..

[84] Zhan J., Gao L., Zhou F., Bai L., Kuang H., He L., Zeng X., Gong H. (2016). Amplitude of low-frequency fluctuations in multiple-frequency bands in acute mild traumatic brain injury. Front. Hum. Neurosci..

[85] Criscuolo A., Schwartze M., Nozaradan S., Kotz S.A. (2025). Basal ganglia and cerebellar lesions causally impact the neural encoding of temporal regularities. Imaging Neurosci. (Camb).

[86] Shirahige L., Berenguer-Rocha M., Mendonça S., Rocha S., Rodriquez M.C., Monte-Silva K. (2020). Quantitative electroencephalography characteristics for Parkinson’s disease: A systematic review. J. Park. Dis..

[87] Tikka S.K., Garg S., Sinha V.K., Nizamie S.H., Goyal N. (2015). Resting state dense array gamma oscillatory activity as a response marker for cerebellar-repetitive transcranial magnetic stimulation (rTMS) in schizophrenia. J. ECT.

[88] Tagawa M., Takei Y., Kato Y., Suto T., Hironaga N., Ohki T., Fukuda M. (2022). Disrupted local beta band networks in schizophrenia revealed through graph analysis: A magnetoencephalography study. Psychiat. Clin. Neuros..

[89] van der Vinne N., Vollebregt M.A., Rush A.J., Eebes M., van Putten M.J.A.M., Arns M. (2021). EEG biomarker informed prescription of antidepressants in MDD: A feasibility trial. ECNP.

[90] Fitzgerald P.J., Watson B.O. (2018). Gamma oscillations as a biomarker for major depression: An emerging topic. Transl. Psychiatry.

[91] De Pascalis V., Vecchio A., Cirillo G. (2020). Resting anxiety increases EEG delta-beta correlation: Relationships with the reinforcement sensitivity theory personality traits. Pers. Ind. Diff..

[92] Coben R., Clarke A.R., Hudspeth W., Barry R.J. (2008). EEG power and coherence in autistic spectrum disorder. Clin. Neurophysiol..

[93] Chouchou F., Khoury S., Chauny J.M., Denis R., Lavigne G.J. (2014). Postoperative sleep disruptions: A potential catalyst of acute pain?. Sleep Med. Rev..

[94] Krenk L., Jennum P., Kehlet H. (2012). Sleep disturbances after fast track hip and knee arthroplasty. Br. J. Anaesth..

[95] Pedersen J.L., Lillesø J., Hammer N.A. (2004). Thiopental and propofol affect different regions of the brain with similar pharmacologic effects. Anesth. Analg..

[96] Shamir M., Goelman G., Chai O. (2008). Postanesthetic cerebellar dysfunction in cats. J. Vet. Intern. Med..

[97] Backeljauw B., Holland S.K., Altaye M., Loepke A.W. (2015). Cognition and brain structure following early childhood surgery with anesthesia. Pediatrics.

[98] Trapani G., Altomare C., Liso G., Sanna E., Biggio G. (2000). Propofol in anesthesia. Mechanism of action, structure-activity relationships, and drug delivery. Curr. Med. Chem..

[99] Bošnjak Ž.J., Logan S., Liu Y., Bai X. (2016). Recent insights into molecular mechanisms of Propofol-induced developmental neurotoxicity: Implications for the protective strategies. Anesth. Analg..

[100] Xiao R., Yu D., Li X., Huang J., Jing S., Bao X., Yang T., Fan X. (2017). Propofol exposure in early life induced developmental impairments in the mouse cerebellum. Front. Cell Neurosci..

[101] Jin R., Liu H., Jin W.-Z., Shi J.-D., Jin Q.-H., Chu C.-P., Qiu D.-L. (2015). Propofol depresses cerebellar Purkinje cell activity via activation of GABA(A) and glycine receptors in vivo in mice. Eur. J. Pharmacol..

[102] Zhang X.-Y., Zhang Y.-D., Cui B.-R., Jin R., Chu C.-P., Jin X.-H., Qiu D.-L. (2020). Propofol facilitates climbing fiber-Purkinje cell synaptic transmission via NMDA receptor in vitro in mice. Eur. J. Pharmacol..

[103] Sachdeva B., Sachdeva P., Ghosh S., Ahmad F., Sinha J.K. (2023). Ketamine as a therapeutic agent in major depressive disorder and posttraumatic stress disorder: Potential medicinal and deleterious effects. Ibrain.

[104] Rooks V.J., Chung T., Connor L., Zurakowski D., Hoffer F.A., Mason K.P., Burrows P.E. (2003). Comparison of oral pentobarbital sodium (nembutal) and oral chloral hydrate for sedation of infants during radiologic imaging: Preliminary results. Am. J. Roentgenol..

[105] Philip A.B., Brohan J., Goudra B. (2005). The role of GABA receptors in anesthesia and sedation: An updated review. CNS Drugs.

[106] Schüttler J., Schwilden H. (2008). Modern Anesthetics.

[107] Chang C.Y., Goldstein E., Agarwal N., Swan K.G. (2015). Ether in the developing world: Rethinking an abandoned agent. BMC Anesthesiol..

[108] Delgado-Herrera L., Ostroff R.D., Rogers S.A. (2001). Sevoflurane: Approaching the ideal inhalational anesthetic: A pharmacologic, pharmacoeconomic, and clinical review. CNS Drug. Rev..

[109] Brosnan R.J., Thiesen R. (2012). Increased NMDA receptor inhibition at an increased Sevoflurane MAC. BMC Anesthesiol..

[110] Eckle V.S., Hauser S., Drexler B., Antkowiak B., Grasshoff C. (2013). Opposing actions of sevoflurane on GABAergic and glycinergic synaptic inhibition in the spinal ventral horn. PLoS ONE.

[111] Ge Y., Ming L., Xu D. (2024). Sevoflurane-induced cognitive effect on α7-nicotine receptor and M1 acetylcholine receptor expression in the hippocampus of aged rats. Neurol. Res..

[112] Hang L.H., Shao D.H., Wang H., Yang J.P. (2010). Involvement of 5-hydroxytryptamine type 3 receptors in sevoflurane-induced hypnotic and analgesic effects in mice. Pharmacol. Rep..

[113] Yukina G.Y., Sukhorukova E.G., Belozertseva I.V., Polushin Y.S., Tomson V.V., Polushi A.Y. (2019). Cerebellar cortex neurons and microglia reaction to Sevoflurane anesthesia. Cell Tiss. Biol..

[114] Fang H., Wang Z.-H., Bu Y.-J., Yuan Z.-J., Wang G.-Q., Guo Y., Cheng X.-Y., Qiu W.-J. (2018). Repeated inhalation of sevoflurane inhibits the information transmission of Purkinje cells and delays motor development via the GABAA receptor ε subunit in neonatal mice. Mol. Med. Rep..

[115] Cassu R.N., Melchert A., Canoa J.T.B., Martins P.D.O. (2014). Sedative and clinical effects of the pharmacopuncture with xylazine in dogs. Acta Cir. Bras..

[116] Kitano T., Kobayashi T., Yamaguchi S., Otsuguro K.I. (2019). The α2A-adrenoceptor subtype plays a key role in the analgesic and sedative effects of xylazine. J. Vet. Pharmacol. Ther..

[117] Ordek G., Groth J.D., Sahin M. (2013). Differential effects of ketamine/xylazine anesthesia on the cerebral and cerebellar cortical activities in the rat. J. Neurophysiol..

[118] de la Peña J.B., Cheong J.H. (2016). The abuse liability of the NMDA receptor antagonist-benzodiazepine (tiletamine-zolazepam) combination: Evidence from clinical case reports and preclinical studies. Drug Test. Anal..

[119] Haigh J.C., Stirling I., Broughton E. (1985). Immobilization of polar bears (Ursus maritimus Phipps) with a mixture of tiletamine hydrochloride and zolazepam hydrochloride. J. Wildl. Dis..

[120] Wilson M.A. (1996). GABA physiology: Modulation by benzodiazepines and hormones. Crit. Rev. Neurobiol..

[121] Mondino A., González J., Li D., Mateos D., Osorio L., Cavelli M., Castro-Nin J.P., Serantes D., Costa A., Vanini G. (2024). Urethane anesthesia exhibits neurophysiological correlates of unconsciousness and is distinct from sleep. Eur. J. Neurosci..

[122] Hara K., Harris R.A. (2002). The anesthetic mechanism of urethane: The effects on neurotransmitter-gated ion channels. Anesth. Analg..

[123] Huang J.J., Yen C.T., Tsao H.W., Tsai M.L., Huang C. (2014). Neuronal oscillations in Golgi cells and Purkinje cells are accompanied by decreases in Shannon information entropy. Cerebellum.

[124] Hermann B.P., Bayless K., Hansen R., Parrish J., Seidenberg M. (2005). Cerebellar atrophy in temporal lobe epilepsy. Epilepsy Behav..

[125] Beckinghausen J., Ortiz-Guzman J., Lin T., Bachman B., Salazar Leon L.E., Liu Y., Heck D.H., Arenkiel B.R., Sillitoe R.V. (2023). The cerebellum contributes to generalized seizures by altering activity in the ventral posteromedial nucleus. Commun. Biol..

[126] Gornati S.V., Schäfer C.B., Eelkman Rooda O.H.J., Nigg A.L., De Zeeuw C.I., Hoebeek F.E. (2018). Differentiating Cerebellar Impact on Thalamic Nuclei. Cell Rep..

[127] Liu R.S.N., Lemieux L., Bell G.S., Bartlett P.A., Sander J.W.A.S., Sisodiya S.M., Schorvon S.D., Duncan J.S. (2001). A longitudinal quantitative MRI study of community-based patients with chronic epilepsy and newly diagnosed seizures: Methodology and preliminary findings. NeuroImage.

[128] Nie L., Jiang Y., Lv Z., Pang X., Liang X., Chang W., Li J., Zheng J. (2022). Deep cerebellar nuclei functional connectivity with cerebral cortex in temporal lobe epilepsy with and without focal to bilateral tonic-clonic seizures: A resting-state fMRI study. Cerebellum.

[129] Sato K., Nakahara K., Obata K. (2022). Hyperperfusion in the cerebellum lobule VIIb in patients with epileptic seizures. BMC Neurol..

[130] Bonilha L., Elm J.J., Edwards J.C., Morgan P.S., Hicks C., Lozar C., Rumboldt Z., Roberts D.R., Rorden C., Eckert M.A. (2010). How common is brain atrophy in patients with medial temporal lobe epilepsy?. Epilepsia.

[131] Nadeau Y., Desbiens R. (2009). The EEG correlates of cerebellar fits. Can. J. Neurol. Sci..

[132] Shouse M.N., Sterman M.B. (1979). Changes in seizure susceptibility, sleep time and sleep spindles following thalamic and cerebellar lesions. Electroenceph. Clin. Neurophysiol..

[133] Dow R.S., Fernández-Guardiola A., Manni E. (1962). The influence of the cerebellum on experimental epilepsy. Electroencephalogr. Clin. Neurophysiol..

[134] Krook-Magnuson E., Szabo G.G., Armstrong C., Oijala M., Soltesz I. (2014). Cerebellar directed optogenetic intervention inhibits spontaneous hippocampal seizures in a mouse model of temporal lobe epilepsy. eNeuro.

[135] Jiang S., Li X., Li Z., Chang X., Chen Y., Huang Y., Zhang Y., Wang H., Zuo X., Li X. (2020). Cerebello-cerebral connectivity in idiopathic generalized epilepsy. Eur. Radiol..

[136] Menon V.B., Kurian J., Undela K., Ramesh M., Gowdappa H.B. (2015). Phenytoin toxicity: A case report. J. Young. Pharm..

[137] Ney G.C., Lantos G., Barr W.B., Schaul N. (1994). Cerebellar atrophy in patients with long-term phenytoin exposure and epilepsy. Arch. Neurol..

[138] Liu R.S., Lemieux L., Bell G.S., Sisodiya S.M., Bartlett P.A., Shorvon S.D., Sander J.W., Duncan J.S. (2005). Cerebral damage in epilepsy: A population-based longitudinal quantitative MRI study. Epilepsia.

[139] Niedermeyer E., Uematsu S. (1974). Electroencephalographic recordings from deep cerebellar structures in patients with uncontrolled epileptic seizures. Electroencephalogr. Clin. Neurophysiol..

[140] Kim J.H., Kim J.B., Suh S.I. (2019). Alteration of cerebello-thalamocortical spontaneous low-frequency oscillations in juvenile myoclonic epilepsy. Acta Neurol. Scand..

[141] Kros L., Eelkman Rooda O.H.J., Spanke J.K. (2015). Cerebellar output controls generalized spike-and-wave discharge occurrence. Ann. Neurol..

[142] Akyuz E., Ozenen C., Pinyazhko O.R., Poshyvak O.B., Godlevsky L.S. (2021). Cerebellar contribution to absence epilepsy. Neurosci. Lett..

[143] Kandel A., Buzsáki G. (1993). Cerebellar neuronal activity correlates with spike and wave EEG patterns in the rat. Epilepsy Res..

[144] Blennow K., Brody D.L., Kochanek P.M., Levin H., McKee A., Ribbers G.M., Yaffe K., Zetterberg H. (2016). Traumatic brain injuries. Nat. Rev. Dis. Prim..

[145] Matis G., Birbilis T. (2008). The Glasgow Coma Scale—A brief review past, present, future. Acta Neurol. Belg..

[146] Andriessen T.M.J.C., Jacobs B., Vos P.E. (2010). Clinical characteristics and pathophysiological mechanisms of focal and diffuse traumatic brain injury. J. Cell. Mol. Med..

[147] Sartor-Glittenberg C., Brickner L. (2014). A multidimensional physical therapy program for individuals with cerebellar ataxia secondary to traumatic brain injury: A case series. Physiother. Theory. Pract..

[148] Drijkoningen D., Leunissen I., Caeyenberghs K., Hoogkamer W., Sunaert S., Duysens J., Swinnen S.P. (2015). Regional volumes in brain stem and cerebellum are associated with postural impairments in young brain-injured patients. Hum. Brain Mapp..

[149] Jang S.H., Kwon H.G. (2017). Injury of the cortico-ponto-cerebellar tract in a patient with mild traumatic brain injury: A case report. Medicine.

[150] Gill-Body K.M., Popat R.A., Parker S.W., Krebs D.E. (1997). Rehabilitation of balance in two patients with cerebellar dysfunction. Phys. Ther..

[151] Caeyenberghs K., Leemans A., Geurts M., Vander Linden C., Smits-Engelsman B.C.M., Sunaert S., Swinnen S. (2011). Correlations between white matter integrity and motor function in traumatic brain injury patients. Neurorehabil. Neural. Repair.

[152] Alexander M.P., Gillingham S., Schweizer T., Stuss D.T. (2012). Cognitive impairments due to focal cerebellar injuries in adults. Cortex.

[153] Kelly R.M., Strick P.L. (2023). Cerebellar loops with motor cortex and prefrontal cortex of a nonhuman primate. J. Neurosci..

[154] Gale S.D., Baxter L., Roundy N., Johnson S.C. (2005). Traumatic brain injury and grey matter concentration: A preliminary voxel-based morphometry study. J. Neurol. Neurosurg. Psychiatry.

[155] Dolenec P., Pilipović K., Janković T., Župan G. (2020). Pattern of neuronal and axonal damage, glial response, and synaptic changes in rat cerebellum within the first week following traumatic brain injury. J. Neuropathol. Exp. Neurol..

[156] Sánchez-Olmedo J.I., Flores-Cordero J.M., Rincón-Ferrari M.D., Péréz-Alé M., Muňoz-Sánchez M.A., Domínquez-Roldán J.M., Murillo-Cabezas F. (2005). Brain death after severe traumatic brain injury: The role of systemic secondary brain insults. Transplant. Proc..

[157] Kinoshita K. (2016). Traumatic brain injury: Pathophysiology for neurocritical care. J. Intensive Care.

[158] Lighthall J.W., Dixon C.E., Anderson T.E. (1989). Experimental models of brain injury. J. Neurotrauma.

[159] Ai J., Baker A. (2002). Presynaptic hyperexcitability at cerebellar synapses in traumatic injury rat. Neurosci. Lett..

[160] Ordek G., Proddutur A., Santhakumar V., Pfister B.J., Sahin M. (2014). Electrophysiological monitoring of injury progression in the rat cerebellar cortex. Front. Syst. Neurosci..

[161] Wang W., Zhang X., He R. (2023). Gamma frequency entrainment rescues cognitive impairment by decreasing postsynaptic transmission after traumatic brain injury. CNS Neurosci. Ther..

[162] Wang C., Costanzo M.E., Rapp P.E. (2017). Disrupted gamma synchrony after mild traumatic brain injury and its correlation with white matter abnormality. Front. Neurol..

[163] Kelley B.J., Petersen R.C. (2007). Alzheimer’s disease and mild cognitive impairment. Neurol. Clin..

[164] Hampel H., Hardy J., Blennow K., Chen C., Perry G., Hyun Kim S., Villemagne V.L., Aisen P., Vendruscolo M., Iwatsubo T. (2021). The Amyloid-β Pathway in Alzheimer’s Disease. Mol. Psychiatry.

[165] Rawat P., Sehar U., Bisht J., Selman A., Culberson J., Reddy P.H. (2022). Phosphorylated Tau in Alzheimer’s Disease and Other Tautopathies. Int. J. Mol. Sci..

[166] Heneka M.T., van der Flier W.M., Jessen F. (2025). Neuroinflammation in Alzheimer’s disease. Nat. Rev. Immunol..

[167] Huang W.J., Zhang X., Chen W.W. (2016). Role of oxidative stress in Alzheimer’s disease. Biomed. Rep..

[168] Thal D.R., Rüb U., Orantes M., Braak H. (2002). Phases of Aβ-deposition in the human brain and its relevance for the development of AD. Neurology.

[169] Braak H., Braak E., Bohl J., Lang W. (1989). Alzheimer’s disease: Amyloid plaques in the cerebellum. J. Neurol. Sci..

[170] Cole G., Neal J.W., Singhrao S.K., Jasani B., Newman G.R. (1993). The distribution of amyloid plaques in the cerebellum and brain stem in Down’s syndrome and Alzheimer’s disease: A light microscopical analysis. Acta Neuropathol..

[171] Guo C.C., Tan R., Hodges J.R., Hu X., Sami S., Hornberger M. (2016). Network-selective vulnerability of the human cerebellum to Alzheimer’s disease and frontotemporal dementia. Brain.

[172] Jernigan T.L., Archibald S.L., Fennema-Notestine C., Gamst A.C., Stout J.C., Bonner J., Hesselink J.R. (2001). Effects of age on tissues and regions of the cerebrum and cerebellum. Neurobiol. Aging.

[173] Sjöbeck M., Englund E. (2001). Alzheimer’s disease and the cerebellum: A morphologic study on neuronal and glial changes. Dement. Geriatr. Cogn. Disord..

[174] Mavroudis I.A., Fotiou D.F., Adipepe L.F. (2010). Morphological changes of the human Purkinje cells and deposition of neuritic plaques and neurofibrillary tangles on the cerebellar cortex of Alzheimer’s disease. Am. J. Alzheimers Dis. Other. Demen..

[175] Mavroudis I.A., Manani M.G., Petrides F., Petsoglou K., Njau S.D., Costa V.G., Baloyannis S.J. (2013). Dendritic and spinal pathology of the Purkinje cells from the human cerebellar vermis in Alzheimer’s disease. Psychiatr. Danub..

[176] Braskie M.N., Medina L.D., Rodriguez-Agudelo Y., Geschwind D.H., Macias-Islas M.A., Thompson P.M., Cummings J.L., Bookheimer S.Y., Ringman J.M. (2013). Memory performance and fMRI signal in presymptomatic familial Alzheimer’s disease. Hum. Brain Mapp..

[177] McLaren D.G., Sreenivasan A., Diamond E.L., Mitchell M.B., Van Dijk K.R.A., Deluca A.N., O’Brien J.L., Rentz D.M., Sperling R.A., Atri A. (2012). Tracking cognitive change over 24 weeks with longitudinal functional magnetic resonance imaging in Alzheimer’s disease. Neurodegener. Dis..

[178] Thomann P.A., Schläfer C., Seidl U., Dos Santos V., Essig M., Schröder J. (2008). The cerebellum in mild cognitive impairment and Alzheimer’s disease–a structural MRI study. J. Psychiatr. Res..

[179] Olazarán J., Hernández-Tamames J.A., Molina E., García-Polo P., Dobato J.L., Álvarez-Linera J., Martínez-Martín P. (2013). AD Research Unit Investigators. Clinical and anatomical correlates of gait dysfunction in Alzheimer’s disease. J. Alzheimers Dis..

[180] Cheron G., Ristori D., Marquez-Ruiz J., Cebolla A.M., Ris L. (2022). Electrophysiological alterations of the Purkinje cells and deep cerebellar neurons in a mouse model of Alzheimer disease (electrophysiology on cerebellum of AD mice). Eur. J. Neurosci..

[181] Yang L., Yan Y., Li Y., Hu X., Lu J., Chan P., Yan T., Han Y. (2020). Frequency-dependent changes in fractional amplitude of low-frequency oscillations in Alzheimer’s disease: A resting-state fMRI study. Brain Imaging Behav..

[182] Meehan C.E., Schantell M., Springer S.D., Wiesman A.I., Wolfson S.L., O’Neil J., Murman D.L., Bares S.H., May P.E., Johnson C.M. (2023). Movement-related beta and gamma oscillations indicate parallels and disparities between Alzheimer’s disease and HIV-associated neurocognitive disorder. Neurobiol. Dis..

[183] Martać L., Podgorac Kojadinović J., Petković B., Sekulić S., Čapo I. (2015). Spectral and fractal analysis of ECoG in animal model of aluminum intoxication. J. Biotech. Res..

[184] Martać L., Podgorac J., Petković B., Stojadinović G. (2022). Aluminum neurotoxicity and neuroprotection. J. Heavy. Met. Toxic. Dis..

[185] Wiesman A.I., Murman D.L., Losh R.A., Schantell M., Christopher-Hayes N., Johnson H.J., Willet M.P., Wolfson S.L., Losh K.L., Johnson C.M. (2022). Spatially resolved neural slowing predicts impairment and amyloid burden in Alzheimer’s disease. Brain.

[186] Paitel E.R., Nielson K.A. (2023). Cerebellar EEG source localization reveals age-related compensatory activity moderated by genetic risk for Alzheimer’s disease. Psychophysiology.

[187] Armstrong M.J., Okun M.S. (2020). Diagnosis and treatment of Parkinson disease: A review. JAMA.

[188] Marquez S., Shafiul H., Siddiquee M.R., Luca C.C., Mishra V.R., Mari Z., Bai O. (2020). Neural correlates of freezing of gait in Parkinson’s disease: An electrophysiology mini-review. Sec. Mov. Disord..

[189] Bucher D., Squire (2009). Central Pattern Generators in Encyclopedia of Neuroscience.

[190] Sen S., Kawaguchi A., Troung Y., Lewis M.M., Huang X. (2010). Dynamic changes in cerebello-thalamo-cortical motor circuitry during progression of Parkinson’s disease. Neuroscience.

[191] Waninger S., Berka C., Stevanovic Karic M., Korszen S., Mozley P.D., Henchcliffe C., Kang Y., Hasterman J., Mangoubi T., Verma A. (2020). Neurophysiological biomarkers of Parkinson’s disease. J. Park. Dis..

[192] Timmermann L., Gross J., Dirks M., Volkmann J., Freund H.J., Schnitzler A. (2002). The cerebral oscillatory network of parkinsonian resting tremor. Brain.

[193] Bosch T.J., Groth C., Eldridge T.A., Gnimpieba E.Z., Baughm L.A., Singh A. (2021). Altered cerebellar oscillations in Parkinson’s disease patients during cognitive and motor tasks. Neuroscience.

[194] Fremont R., Calderon D.P., Maleki S., Khodakhah K. (2014). Abnormal high-frequency burst firing of cerebellar neurons in rapid-onset dystonia-parkinsonism. J. Neurosci..

[195] Schurrman P.R., Bosch D.A., Bossuyt P.M., Bonselm G.J., van Someren E.J., de Bie R.M., Merkus M.P., Speelman J.D. (2000). A comparison of continuous thalamic stimulation and thalamotomy for suppression of severe tremor. N. Eng. J. Med..

[196] Ferrucci R., Cortese F., Bianchi M., Pittera D., Turrone R., Bocci T., Borroni B., Vergari M., Cogiamanian F., Ardolino G. (2016). Cerebellar and motor cortical transcranial stimulation decrease levodopa-induced dyskinesias in Parkinson’s disease. Cerebellum.

[197] Berman B.D., Smucny J., Wylie K.P., Shelton E., Kronberg E., Leehey M., Tregellas J.R. (2016). Levodopa modulates small-world architecture of functional brain networks in Parkinson’s disease. Mov. Disord..

[198] Mueller K., Jech R., Ballarini T., Holiga Š., Růžička F., Piecha F.A., Möller H.E., Vymazal J., Schroeter M.L. (2019). Modulatory effects of levodopa on cerebellar connectivity in Parkinson’s disease. Cerebellum.

[199] Schmahmann J.D., Caplan D. (2006). Cognition, emotion and the cerebellum. Brain.

[200] Schutter D.J.L.G., van Honk J. (2006). An electrophysiological link between the cerebellum, cognition and emotion: Frontal theta EEG activity to single-pulse cerebellar TMS. Neuroimage.

[201] Elvevag B., Goldberg T.E. (2000). Cognitive impairment in schizophrenia is the core of the disorder. Crit. Rev. Neurobiol..

[202] Buckner R.L., Krienen F.M., Castellanos A., Diaz J.C., Yeo B.T. (2011). The organization of the human cerebellum estimated by intrinsic functional connectivity. J. Neurophysiol..

[203] Courchesne E. (1997). Brainstem, cerebellar and limbic neuroanatomical abnormalities in autism. Curr. Opin. Neurobiol..

[204] Moberget T., Doan N.T., Alnæs D., Kaufmann T., Córdova-Palomera A., Lagerberg T.V., Diedrichsen J., Schwarz E., Zink M., Eisenacher S. (2018). Cerebellar volume and cerebellocerebral structural covariance in schizophrenia: A multisite mega-analysis of 983 patients and 1349 healthy controls. Mol. Psychiatry.

[205] Mavroudis I.A., Petrides F., Manani M., Chatzinikolaou F., Ciobică A.S., Pădurariu M., Kazis D., Njau S.N., Costa V.G., Baloyannis S.J. (2017). Purkinje cells pathology in schizophrenia. A morphometric approach. Rom. J. Morphol. Embryol..

[206] Andreasen N.C., O’Leary D.S., Cizadlo T., Arndt S., Rezai K., Ponto L.L. (1996). Schizophrenia and cognitive dysmetria: A positron-emission tomography study of dysfunctional prefrontal-thalamic-cerebellar circuitry. Proc. Natl. Acad. Sci. USA.

[207] Shin Y.W., O’Donnell B.F., Youn S., Kwon J.S. (2011). Gamma oscillation in schizophrenia. Psychiatry Investig..

[208] Fryer S.L., Roach B.J., Wiley K., Loewy R.L., Ford J.M., Mathalon D.H. (2016). Reduced amplitude of low-frequency brain oscillations in the psychosis risk syndrome and early illness schizophrenia. Neuropsychopharmacology.

[209] Frazier M.R., Hoffman L.J., Popal H., Sullivan-Toole H., Olino T.M., Olson I.R. (2022). A missing link in affect regulation: The cerebellum. Soc. Cogn. Affect. Neurosci..

[210] Stoodley C.J., Schmahmann J.D. (2010). Evidence for topographic organization in the cerebellum of motor control versus cognitive and affective processing. Cortex.

[211] Depping M.S., Wolf N.D., Vasic N., Sambataro F., Hirjak D., Thomann P.A., Wolf R.C. (2016). Abnormal cerebellar volume in acute and remitted major depression. Prog. Neuropsychopharmacol. Biol. Psychiatry.

[212] Chin P.W., Augustine G.J. (2023). The cerebellum and anxiety. Front. Cell. Neurosci..

[213] Lai M.-C., Lombardo M.V., Baron-Cohen S. (2014). Autism. Lancet.

[214] Fatemi S.H., Folsom T.D., Reutiman T.J., Thuras P.D. (2009). Expression of GABAB receptors is altered in brains of subjects with autism. Cerebellum.

[215] Malan N.S., Gopalakrishnan R., Cunningham D., Hogue O., Baker K.B., Machado A.G. (2025). Human Cortico-Cerebellar Dynamics During Motor Error Processing After Stroke. Hum. Brain. Mapp..

[216] Kawato M., Kuroda T., Imamizu H., Nakano E., Miyauchi S., Yoshioka T. (2003). Internal forward models in the cerebellum: fMRI study on grip force and load force coupling. Prog. Brain Res..

[217] Popa L.S., Ebner T.J., Manto M.U., Gruol D.L., Schmahmann J.D., Koibuchi N., Sillitoe R.V. (2022). Cerebellum and Internal Models. Handbook of the Cerebellum and Cerebellar Disorders.

[218] Mangalam M. (2025). The illusion of internal models in biological movement. Eur. J. Appl. Physiol..

[219] Manto M., Mitoma H. (2025). Cerebellar syndromes: Clinical observations leading to the recognition of the three types. Arq. Neuropsiquiatr..

[220] Schmahmann J.D. (2004). Disorders of the cerebellum: Ataxia, dysmetria of thought, and the cerebellar cognitive affective syndrome. J. Neuropsychiatry Clin. Neurosci..

[221] Bodranghien F., Bastian A., Casali C., Hallett M., Louis E.D., Manto M., Mariën P., Nowak D.A., Schmahmann J.D., Serrao M. (2016). Consensus Paper: Revisiting the Symptoms and Signs of Cerebellar Syndrome. Cerebellum.

[222] Ataullah A.H.M., Singla R., Naqvi I.A. (2024). Cerebellar Dysfunction. StatPearls.

[223] Smith L.A., Olkhova E.A., Lax N.Z., Ng Y.S., Taylor R.W., Gorman G.S., Erskine D., McFarland R. (2025). Delineating the mechanisms of cerebellar degeneration in pediatric and adult primary mitochondrial disease. Acta Neuropathol..

[224] Lin C.R., Kuo S.H. (2023). Ataxias: Hereditary, Acquired, and Reversible Etiologies. Semin. Neurol..

[225] Parvez M.S.A., Ohtsuki G. (2022). Acute Cerebellar Inflammation and Related Ataxia: Mechanisms and Pathophysiology. Brain Sci..

[226] Wang J., Zhou X.-L., Ma Z.-H., Liu L., Zhou Q., Wen J.-W., Wen J.-H., Su H., Zhang Y.-H., Xia X.-C. (2025). Cerebellar Mechanisms Underlying Autism-like Cognitive Deficits in Mouse Offspring with Prenatal Valproic Acid Exposure. Toxics.

[227] Sabo S.L., Lahr J.M., Offer M., Weekes A.L.A., Sceniak M.P. (2023). GRIN2B-related neurodevelopmental disorder: Current understanding of pathophysiological mechanisms. Front. Synaptic Neurosci..

[228] Reeber S.L., Otis T.S., Sillitoe R.V. (2013). New roles for the cerebellum in health and disease. Front. Syst. Neurosci..

[229] Manto M., Marmolino D. (2009). Cerebellar Disorders—At the Crossroad of Molecular Pathways and Diagnosis. Cerebellum.

[230] Khatun M.M., Biswas M.S., Podder M.K., Hasan R., Siddika M.A. (2026). The cerebro-cerebellar system: Integrative roles in motor control, cognition, and neuropsychiatric disorders. IBRO Neurosci. Rep..

[231] Millett D. (2001). Hans Berger: From psychic energy to the EEG. Perspect. Biol. Med..

[232] Sysoev Y.I., Okovityi S.V. (2024). Prospects of Electrocorticography in Neuropharmacological Studies in Small Laboratory Animals. Brain Sci..

[233] Niso G., Krol L.R., Combrisson E., Dubarry A.S., Elliott M.A., François C., Héjja-Brichard Y., Herbst S.K., Jerbi K., Kovic V. (2022). Good scientific practice in EEG and MEG research: Progress and perspectives. Neuroimage.

[234] Koessler L. (2023). What Are the Advantages and Challenges of Simultaneous Scalp EEG and Intracranial EEG Data Recording?. Intracranial EEG: A Guide for Cognitive Neuroscientists.

[235] Parvizi J., Kastner S. (2018). Promises and limitations of human intracranial electroencephalography. Nat. Neurosci..

[236] Merk T., Peterson V., Lipski W.J., Blankertz B., Turner R.S., Li N., Horn A., Richardson R.M., Neumann W.J. (2022). Electrocorticography is superior to subthalamic local field potentials for movement decoding in Parkinson’s disease. eLife.

[237] Han Y., Oh S.S., Kang J.K., Park H. (2014). Simultaneous Measurement of fMRI and EEG—Principles and Applications. Advanced Brain Neuroimaging Topics in Health and Disease-Methods and Applications.

[238] Yen C., Lin C.-L., Chiang M.-C. (2023). Exploring the Frontiers of Neuroimaging: A Review of Recent Advances in Understanding Brain Functioning and Disorders. Life.

[239] Friston K.J., Fletcher P., Josephs O., Holmes A., Rugg M.D., Turner R. (1998). Event-related fMRI: Characterizing differential responses. Neuroimage.

[240] Schrooten M., Vandenberghe R., Peeters R., Dupont P. (2019). Quantitative Analyses Help in Choosing Between Simultaneous vs. Separate EEG and fMRI. Front Neurosci..

[241] Sotero R.C., Trujillo-Barreto N.J. (2008). Biophysical model for integrating neuronal activity, EEG, fMRI and metabolism. Neuroimage.

